# Mechanisms and targeted prevention of abnormal ductular reaction caused by a low concentration of Benzo(a)pyrene

**DOI:** 10.1038/s41419-025-08043-8

**Published:** 2025-10-07

**Authors:** Xinru Du, Yancheng Gao, Sisi Song, Qinming Hui, Zhendong Wang, Mengyue Ji, Maoxuan Li, Shuoke Duan, Sha Liu, Ziyi Wang, Yue Ma, Ye Yang, Chunxiao Zhou, Yuan Li

**Affiliations:** 1 Department of Gastroenterology, The Affiliated Suzhou Hospital of Nanjing Medical University, Suzhou, Jiangsu Province China; 2 The Key Laboratory of Modern Toxicology, Ministry of Education, School of Public Health, Nanjing Medical University, Nanjing, Jiangsu Province China; 3 School of Public Health, Key Laboratory of Public Health Safety and Emergency Prevention and Control Technology of Higher Education Institutions in Jiangsu Province, Nanjing Medical University, Nanjing, Jiangsu Province China

**Keywords:** Mechanisms of disease, Epigenetics

## Abstract

The impact of long-term exposure to low concentrations of environmental pollutants on hepatobiliary diseases is a major public health issue. Benzo(a)pyrene (B[a]P) is a common environmental toxin classified by the International Agency for Research on Cancer as a Group I carcinogen. Abnormal ductular reaction (DR) is a major pathological feature of hepatobiliary diseases; however, the underlying molecular mechanisms of B[a]P-induced abnormal DR remain unclear. This study revealed that chronic exposure to a low concentration of B[a]P increased the expression of a glucose-regulated protein (GRP75) in cholangiocytes. As GRP75 is a bridge protein for the endoplasmic reticulum (ER)-mitochondrial junction, the overexpression of GRP75 abnormalizes ER-mitochondria coupling. These biological processes facilitated Ca^2+^ release from the ER into the mitochondria and caused mitochondrial Ca^2+^ overload, leading to the overproduction of reactive oxygen species (ROS). The increased ROS activates epithelial-mesenchymal transition (EMT) and ultimately induces a profibrotic phenotype in bile duct cells (BECs). These cells secreted collagen and activated hepatic stellate cells through paracrine activity, which synergistically promoted the development and progression of fibrosis. Finally, via drug screening and functional analysis, we innovatively revealed a traditional Chinese medicine monomer, luteolin, which could prevent B[a]P-induced abnormal DR and hepatic fibrosis by targeting GRP75. Our study offers new insights into environmental toxin-induced hepatobiliary diseases and suggests a potential key interventional target or approach for the prevention of abnormal DR.

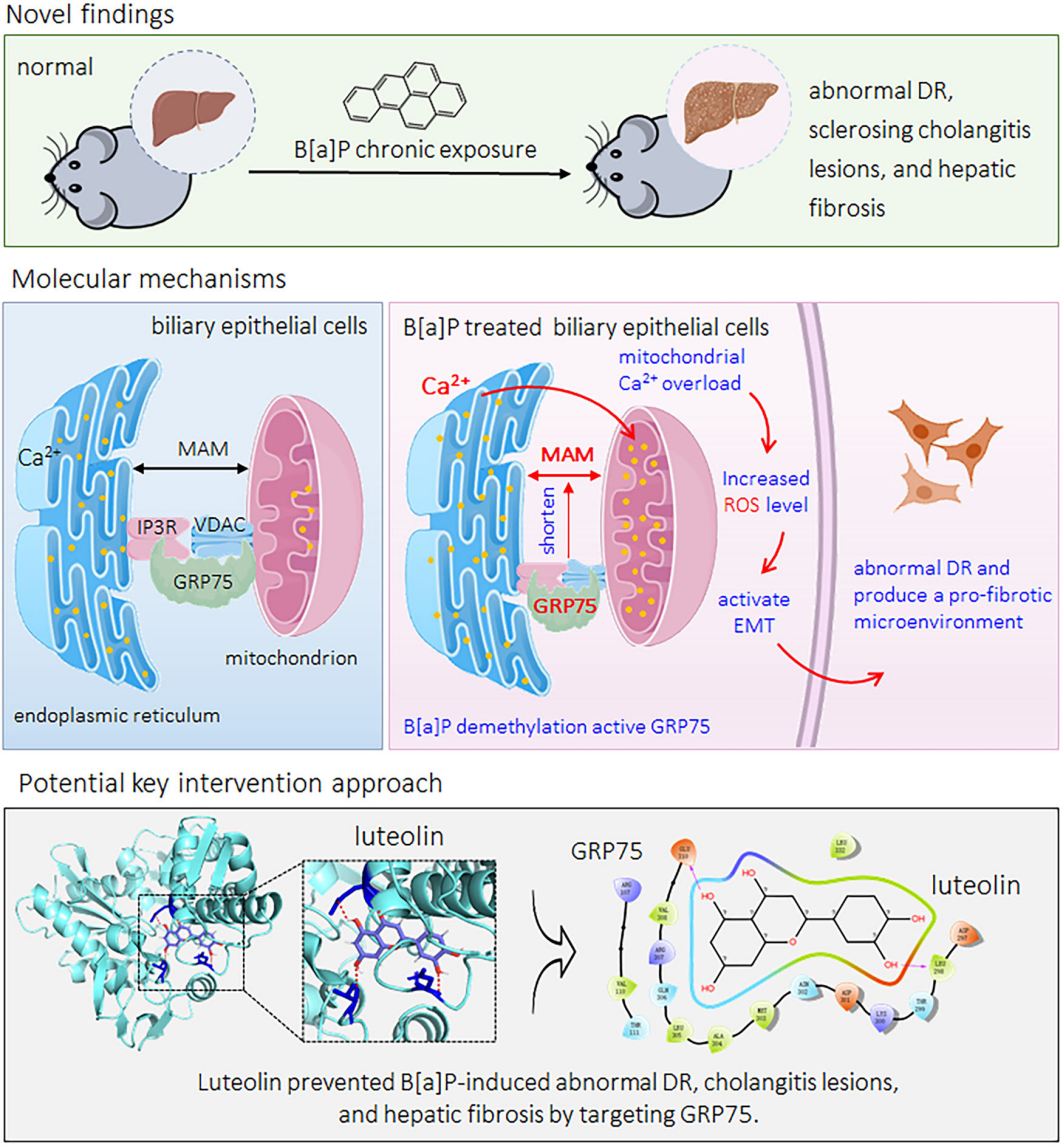

## Introduction

Ductal reaction (DR) is a common injury repair response in many hepatobiliary diseases and is histologically characterized by reactive bile duct hyperplasia [1]. The dominant feature of DR is hyperplasia of reactive-appearing ductlike cells, including hepatic progenitor cells (HPCs), cholangiocytes and hepatocytes, the proliferation of which contributes to the development of DR [2, 3]. Abnormal DR is a characteristic pathological change in hepatobiliary diseases, such as sclerosing cholangitis and hepatic fibrosis [4, 5]. Without early intervention, abnormal DR may deteriorate and eventually lead to cirrhosis and even hepatocellular carcinoma (HCC) and cholangiocarcinoma (CCA) [6].

Epidemiological studies have identified toxic exposure and poor lifestyle choices as the main causes of chronic hepatobiliary diseases, with environmental toxic exposure being one of the most representative risk factors [7, 8]. Benzo(a)pyrene (B[a]P) is a common environmental toxin classified by the International Agency for Research on Cancer (IARC) as a Group I carcinogen and is formed by the incomplete combustion of organic materials in many industrial and food-cooking processes [9, 10]. After it enters the body, the absorbed B[a]P is mainly distributed in the liver, which contains several enzymes essential for the bioactivation of B[a]P. Therefore, compared with other organs, B[a]P is more toxic to the liver [11, 12]. Our previous research revealed that B[a]P promotes multidrug resistance in HCC [13]. Current research has focused mainly on the toxic effects of B[a]P on hepatocytes; however, the relationship between B[a]P and DR remains inadequately explored.

Glucose-regulated protein 75 (GRP75) is a unique member of the HSP70 family located predominantly in the mitochondrial matrix that regulates a variety of cellular processes, including cell survival, growth and metabolism [14]. In addition, GRP75 is thought to bridge mitochondria and the endoplasmic reticulum (ER) through the assembly of the inositol triphosphate receptor (IP3R)-GRP75-voltage-dependent anion channel (VDAC) complex and play a crucial role in regulating the distance and interaction between mitochondria and the ER [15]. GRP75, an important mitochondrial chaperone, is closely associated with the development of various diseases, such as CCA, hepatic lipogenesis and colon cancer [16–18]. Our latest study revealed that GRP75 plays a key role in the B[a]P-promoted progression of HCC [19]. However, the effects of GRP75 on B[a]P-induced abnormal DR and the underlying molecular mechanisms remain unclear. This study aimed to explore the molecular mechanisms of GRP75 in B[a]P exposure-induced abnormal DR in cellular and animal models through a variety of molecular biology techniques and to investigate novel potential targeted intervention approaches.

## Materials and methods

### Reagents and drugs

B[a]P (C_20_H_12_, >96.0% purity) and 3,5-diethoxycarbonyl-1,4-dihydrocollidine (DDC, C_14_H_21_NO_4_, 99.0% purity) were purchased from Sigma-Aldrich China Co (Shanghai, China). Corn oil and luteolin (C_15_H_10_O_6_, >99.5%purity) were purchased from MedChemExpress (Shanghai, China). All other reagents were of analytical grade or the highest grade available.

### Mice and in vivo treatment

This study was approved by the Nanjing Medical University Institutional Animal Care and Use Committee (the permit No. IACUC-2209058), and the animals were treated humanely to alleviate suffering. Male C57BL/6 J mice (8 weeks old, 25 ± 2 g) were purchased from the Animal Laboratory Center of Nanjing Medical University. All mice were kept under 12 h:12 h dark/light cycle at a consistent temperature (25 °C) and provided with standard chow with access to sterile water freedom. The NC group was administered 0.1 mL of corn oil for 4 weeks. In the B[a]P treatment group, mice were administered B[a]P via oral gavage at a dose of 12.5 mg/kg (dissolved in corn oil) daily for 4 weeks [20]. In the DDC (served as a positive control) treatment group, mice were fed with containing 0.1% DDC for 4 weeks. For the prevention model, mice were treated with luteolin by oral gavage at a dose of 10 mg/kg, daily for 4 weeks [21]. After treatment, all mice were euthanized simultaneously, and the liver tissue and serum were subjected to further investigation.

### Cells and cell culture

For the isolation of primary bile duct cells (BECs), the entire procedure was conducted on a sterile operating table. C57BL/6 J mice were euthanized under anesthesia with pentobarbital sodium. An abdominal midline incision was made to expose the liver, which was then perfused with 1× HEPES-buffered saline containing 0.02% (wt/vol) egtazic acid (Sigma-Aldrich) until pale, indicating effective blood clearance. Perfusion continued using 0.02% (wt/vol) collagenase (Sigma-Aldrich). Subsequently, the liver was removed and placed in a sterile 10 cm dish containing ice-cold DMEM/F12 medium (Gibco, Grand Island, NY). After removing the liver peritoneum, it was placed in collagenase IV (MedChemExpress, Shanghai, China) digestive solution and digested at 37 °C on a shaker for 10 min. The liver parenchymal cells were brushed away to extract the biliary tree. The bile duct was cut into pieces and digested with prewarmed 0.5 g/L collagenase I and 4.2 g/L dispase (Gibco) solution in DMEM/F12 medium at 37 °C for 15 min. The suspension was filtered through a 100 μm cell strainer (Corning, NY, USA), and the residual tissue fragments were re-digested. The collected cell suspension was washed by centrifugation, and the primary BECs were aggregated as precipitates [22]. Primary BECs precipitate was resuspended in DMEM/F12 medium to a cell density of 1 × 10^5^/mL with careful pipetting. The cells were inoculated into 25 mL plastic culture flasks coated with type I rat tail collagen (Sigma-Aldrich) and cultured in a 37 °C, 5% CO_2_ incubator [23].

The cholangiocarcinoma cell line (SG231) was obtained from and short tandem repeat identified by Shanghai Honsun Biological Co. Ltd (Shanghai, China); while the human hepatic stellate cell (HSC) line (LX-2) was obtained from and short tandem repeat identified by KeyGen Co. Ltd (Nanjing, China). SG231 cells were cultured in RPMI-1640 medium (Gibco), supplemented with 10% fetal bovine serum (FBS, Gibco), 100 U/mL penicillin (Beyotime Co. Ltd., Shanghai, China), and 100 μg/mL streptomycin (Beyotime). While LX2 cells were cultured in Dulbecco’s Modified Eagle Medium (DMEM, Gibco) supplemented with 10% FBS, 100 U/mL penicillin, and 100 μg/mL streptomycin. The cells were routinely cultured at 37°C in a humidified incubator containing 5% CO_2_.

### Molecular biology experiments

The experimental procedure for cell transfection, DNA methylation analysis, Fluorescence mitochondrial calcium and ER calcium imaging, transmission electron microscopy (TEM), reactive oxygen species (ROS) staining and quantification, quantitative real-time polymerase chain reaction (qRT-PCR), western blot, immunofluorescence staining (IF), histological analyses, immunohistochemistry (IHC), enzyme-linked immunosorbent assay (ELISA), and data mining and bioinformatics analysis please see the detailed information listed item by item in the supplementary materials and methods section.

### Drug screening and functional analysis

The Coremine (https://coremine.com/), high-throughput experiment and reference-based database (HERB, http://herb.ac.cn/) and symptom mapping (SymMap, http://www.symmap.org/) databases were used to identify traditional Chinese medicines (TCMs) with therapeutic effects on cholangitis; the traditional Chinese medicine integrated pharmacology (TCMIP, http://www.tcmip.cn/TCMIP/index.php/) database was used to reveal potentially effective key monomeric chemicals in TCMs; and the PyMOL software was used to simulate molecular docking of chemicals with the GRP75 protein. For the combination of recombinant GRP75 protein and luteolin, as we described previously [24], the protein-ligand interactions were measured by biolayer interferometry (BLI) assay.

### Statistical analysis

Statistical analysis was performed by GraphPad Prism (version 9.0.0 for Windows; San Diego, California, USA; www.graphpad.com). The statistical significance was determined using a two-tailed Student’s *t*-test, or a one-way analysis of variance (ANOVA) followed by Tukey’s *t*-test. The data were presented as mean ± standard deviation (SD). A *p*-value < 0.05 was considered statistically significant.

## Results

### Establishing a low-concentration B[a]P-induced sclerosing cholangitis-like lesion model in C57BL/6 J mice

**Fig. 1:**
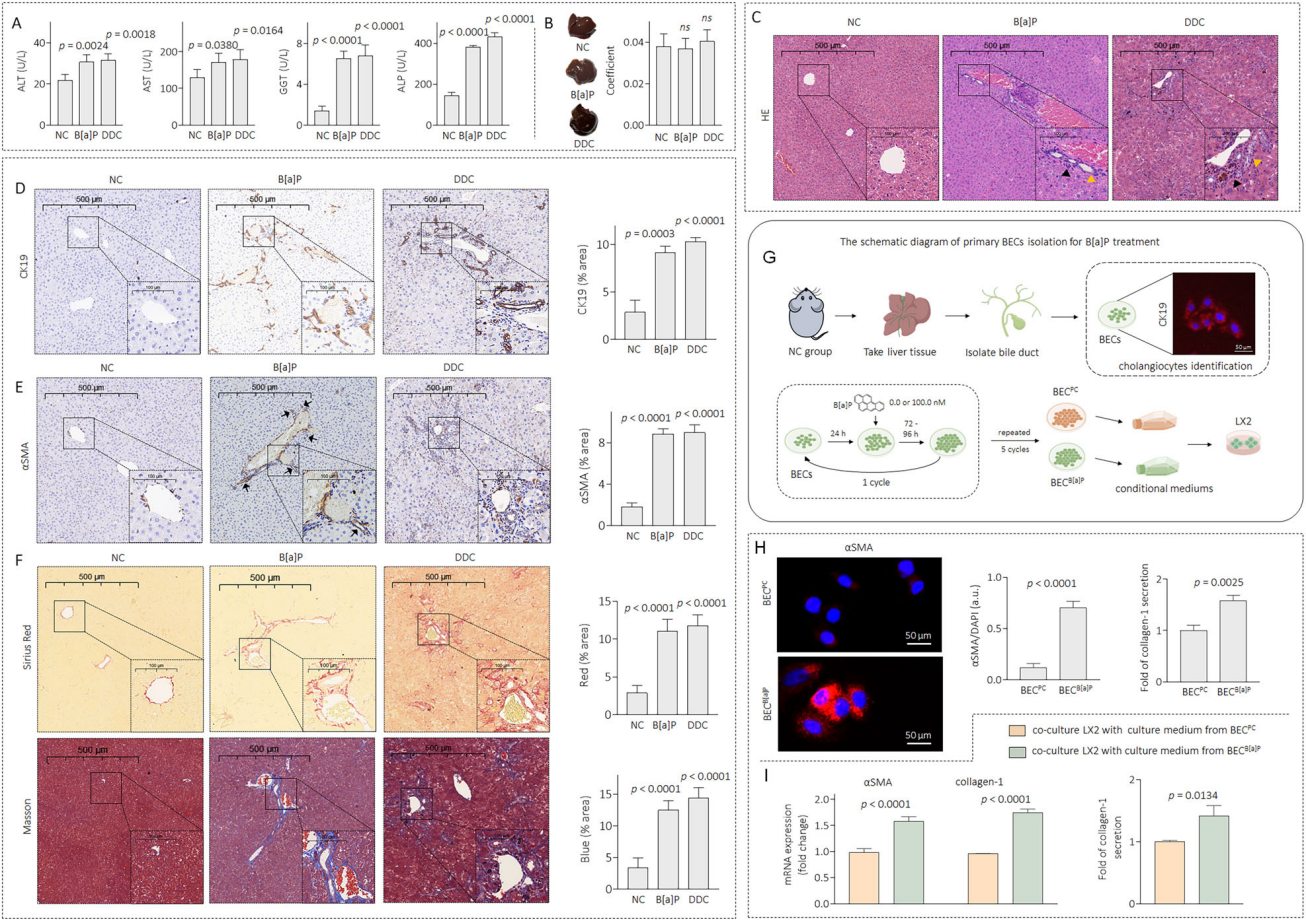
Establishing a low concentration of B[a]P-induced sclerosing cholangitis-like lesion model in C57BL/6 J mice. **A** Biochemical analysis of blood in NC, B[a]P and DDC groups, *n* = 5. **B** Liver gross appearance and coefficient (liver weight/body weight ratio), *n* = 5. **C** Liver sections were stained with H&E (yellow arrows denote bile ducts, black arrows denote inflammatory cell infiltration), *n* = 5. **D**, **E** IHC analysis of CK19 and αSMA, *n* = 5. **F** Liver sections were stained with Sirius Red and Masson, *n* = 5. **G** A sketch map for the isolation and identification of primary BECs. **H** IF staining analysis of αSMA and triplicate ELISA analysis of collagen-1 in BEC^PC^ and BEC^B[a]P^ cells, *n* = 3. **I** Triplicate qPCR/ELISA analysis of αSMA and collagen-1 in LX2 treated by conditioned media, *n* = 3. Data were shown as mean ± SD. An ANOVA followed by Tukey’s *t*-test was used for between-group comparisons (for **A** and **B**, **D** to **F**), and a two-tailed Student’s t-test was used for between-group comparisons (for **H**, **I**).

Male C57BL/6J mice were randomly divided into NC and B[a]P groups; 3,5-diethoxycarbonyl-1,4-dihydrocollidine (DDC) was used as a positive control. After 4 weeks of treatment, compared with those in the NC group, serum levels of ALT, AST, GGT and ALP were significantly increased in the B[a]P and DDC groups (Fig. 1A). No significant differences in liver appearance or coefficients were observed among these groups (Fig. 1B); however, histological examination of liver tissues from the B[a]P and DDC groups revealed irregular biliary proliferation in the portal area accompanied by pronounced inflammatory cell infiltration (Fig. 1C). Moreover, in these two groups, the reactive bile ducts were markedly hyperplastic (as determined by CK19 and αSMA staining, Fig. 1D, E). In addition, massive collagen deposition and a typical onion skin-type change (as determined by Masson and Sirius red staining) were observed in the B[a]P and DDC groups (Fig. 1F).

During chronic liver injury, HPCs are activated to proliferate and differentiate into cholangiocytes and hepatocytes, leading to aberrant bile duct hyperplasia and hepatic fibrosis [2]. However, it remains unclear whether cholangiocytes act as target cells upon B[a]P exposure. Here, we isolated primary BECs (CK19-positive cells) from NC-group mice and applied 0.0 or 100.0 nM B[a]P for 6 culture cycles (labelled BEC^PC^ and BEC^B[a]P^, respectively) to mimic the biological alterations caused by long-term low-dose B[a]P exposure (Fig. 1G). Compared with BEC^PC^ cells, BEC^B[a]P^ cells had markedly increased αSMA expression and collagen-1 secretion (Fig. 1H). Moreover, the conditioned medium collected from BEC^B[a]P^ cells dramatically activated hepatic stellate cells (LX2 cells), as determined by increased expression and secretion of αSMA and collagen-1 (Fig. 1I). We also confirmed our data in another cell line, SG231 (a cholangiocarcinoma cell line typically used in studies related to DR [25], Supplementary Fig. S1). Collectively, these results indicated that long-term exposure to a low concentration of B[a]P caused abnormal DR, and BEC/SG231^B[a]P^ cells secreted collagen and activated hepatic stellate cells through paracrine action, which synergistically promoted the development and progression of sclerosing cholangitis-like pathological lesions.

### Low-dose B[a]P exposure continuously activated GRP75 via the epigenetic accumulation of DNA demethylation

**Fig. 2:**
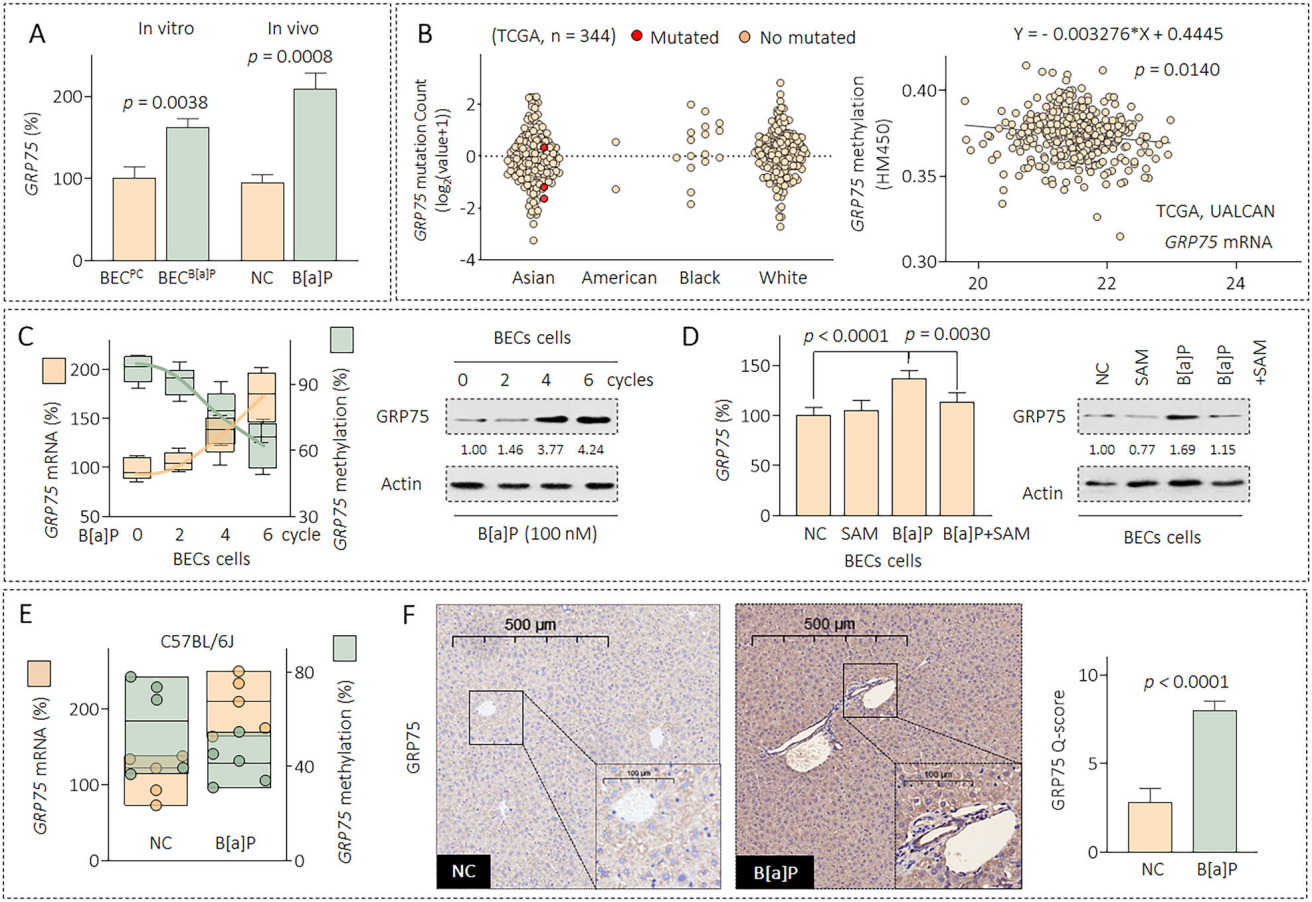
Low-dose B[a]P exposure continuously activated GRP75 via the epigenetic accumulation of DNA demethylation. **A** Triplicate qPCR analysis of GRP75. **B** A mutation analysis (left), and the mRNA and methylation levels (right) of GRP75 in liver cancer patients based on the TCGA database. **C** Triplicate qMSP/qPCR (left) and Western blot analysis (right) of GRP75 expression or promoter methylation, *n* = 3. **D** After BECs were treated by 0.0 or 100.0 nM of B[a]P in the presence or absence of 100.0 μm S-adenosylmethionine (SAM, a methyl donor) for 96 h, triplicate qMSP/qPCR (left) and Western blot analysis (right) of GRP75 expression or promoter methylation were performed, *n* = 3. **E**, **F** Triplicate qMSP/qPCR and IHC analysis of GRP75 expression or promoter methylation in mouse liver sections, *n* = 5. Data were shown as mean ± SD. An ANOVA followed by Tukey’s *t*-test was used for between-group comparisons (for **D**), and a two-tailed Student’s t-test was used for between-group comparisons (for **A**, **F**).

Our previous studies demonstrated that GRP75 is involved in the development and progression of B[a]P-induced hepatocellular carcinoma [19]. In this study, the expression of GRP75 was notably elevated in cells and mice following long-term exposure to a low concentration of B[a]P (Fig. 2A). The synergistic regulation of genetic and epigenetic mechanisms is involved in the hazardous effects induced by environmental pollutants [20]. In this study, based on the TCGA database, the GRP75 gene was mutated very infrequently in HCC; nevertheless, we detected a negative correlation between the GRP75 mRNA and GRP75 promoter methylation levels (Fig. 2B). DNA methylation is an important epigenetic modification in various diseases induced by B[a]P exposure [20]. Here, BECs undergoing prolonged exposure to B[a]P demonstrated a gradual increase in GRP75 mRNA and protein levels and a corresponding decrease in GRP75 promoter methylation (Fig. 2C). Additionally, after BECs were treated with 0 or 100 nM B[a]P in the presence or absence of the methyl donor S-adenosylmethionine (SAM), we observed that SAM markedly suppressed the B[a]P-induced increase in GRP75 (Fig. 2D). Furthermore, in the liver tissues of the B[a]P group, compared with those of the NC group, higher expression levels of GRP75 mRNA and protein and a lower methylation level at the GRP75 promoter were observed (Fig. 2E, F). We also confirmed our data in another cell line, SG231 (supplementary Fig. S2). Interestingly, as shown in Fig. 2F, exposure to B[a]P significantly upregulated the protein expression of GRP75 in both hepatocytes and BECs. Furthermore, immunofluorescence confirmed that the expression levels of GRP75 in both cell types were significantly elevated in the B[a]P-treated group (supplementary Fig. S3). Our previous study revealed that B[a]P could increase the GRP75 expression in HCC cells [13]. However, the effects and mechanisms of B[a]P and GRP75 in BECs remain largely uninvestigated. In this study, we found that GRP75 was gradually activated by the epigenetic accumulation of DNA demethylation in BECs following chronic exposure to a low concentration of B[a]P.

### B[a]P causes abnormal ER-mitochondrial coupling, mitochondrial Ca^2+^ overload, excessive ROS production, and epithelial-mesenchymal transition (EMT) via GRP75

**Fig. 3:**
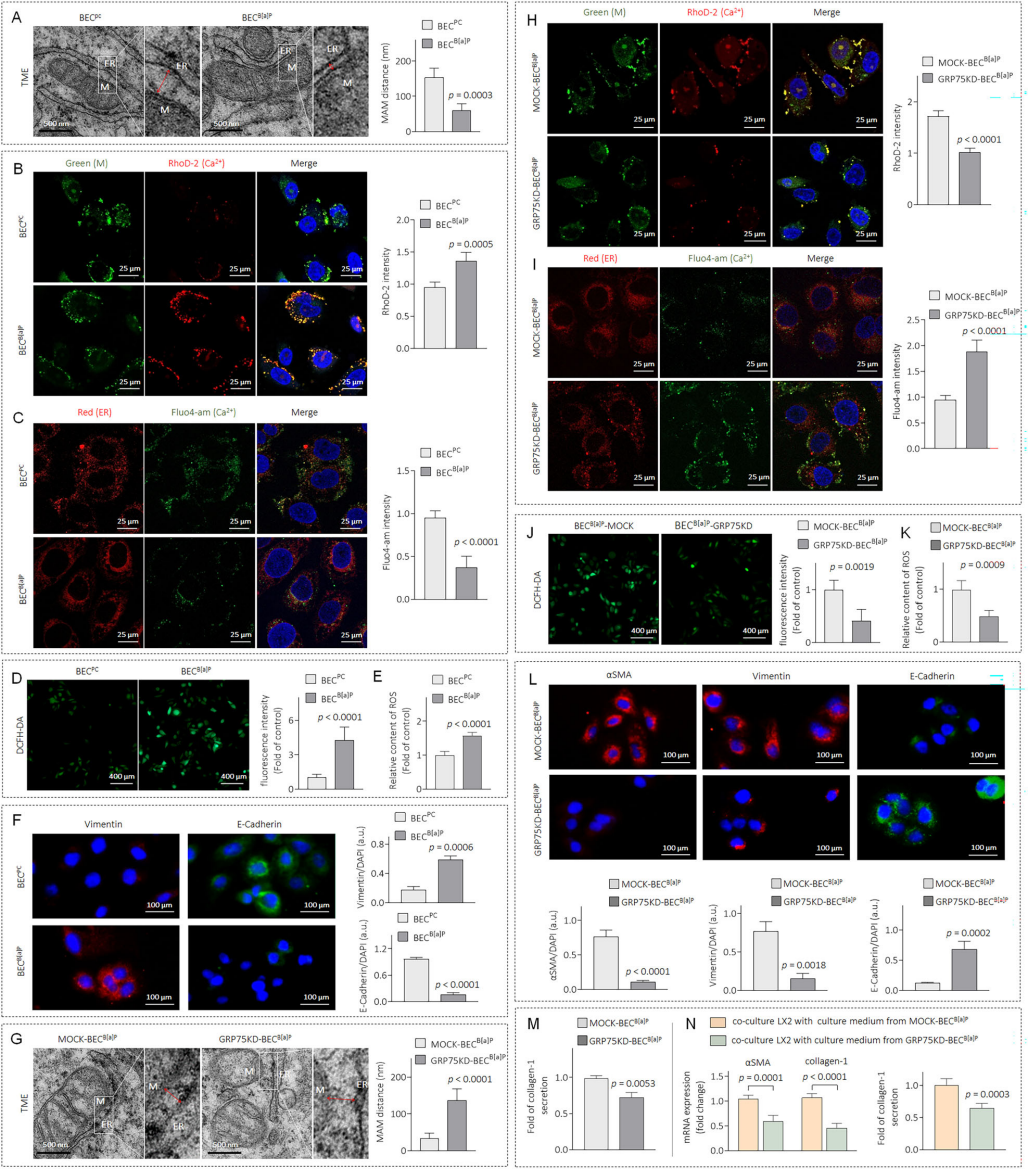
B[a]P causes abnormal ER-mitochondrial coupling, mitochondrial Ca^2+^ overload, excessive ROS production, and EMT via GRP75. **A**–**E** In BEC^PC^ and BEC^B[a]P^ cells. **A** TEM image (note: M: mitochondrial; ER: endoplasmic reticulum). **B** IF staining analysis of mitochondrial and Ca^2+^ co-localisation. **C** IF staining analysis of ER and Ca^2+^ co-localisation. **D**, **E** Intracellular ROS levels by DCFH-DA fluorescence and multi-well plate reader detection. **F** IF staining analysis of vimentin and E-Cadherin. **G**–**M** BEC^B[a]P^ cells were transfected by NC- or GRP75-siRNA. **G** TEM images. **H** IF staining analysis of mitochondrial and Ca^2+^ co-localisation. **I** IF staining analysis of ER and Ca^2+^ co-localisation. **J**, **K** Intracellular ROS levels. **L** IF staining analysis of αSMA, vimentin and E-Cadherin. **M** Triplicate ELISA analysis of collagen-1. **N** After LX2 cells were treated by conditioned media collected from MOCK-BEC^B[a]P^ or GRP75-KD-BEC^B[a]P^ cells, triplicate qPCR/ELISA analysis of αSMA or collagen-1 was performed. Data was shown as mean ± SD, *n* = 3, a two-tailed Student’s *t*-test was used for between-group comparisons.

GRP75 is an important protein on the mitochondria-associated endoplasmic reticulum membrane, and its aberrant expression leads to abnormal ER-mitochondrial coupling [14]. As shown in Fig. 3A, BEC^B[a]P^ cells exhibited abnormal ER-mitochondrial coupling, characterized by a remarkable shortening of the contact distance between the organelles. The decreased distance between the ER and the mitochondria facilitates the flow of Ca^2+^ from the endoplasmic reticulum to the mitochondria, leading to mitochondrial Ca^2+^ overload and massive ROS production [26]. We verified that the mitochondrial Ca^2+^ concentration significantly increased in BEC^B[a]P^ cells, whereas the Ca^2+^ concentration in the endoplasmic reticulum relatively decreased (Fig. 3B, C). Moreover, compared with those in BEC^PC^ cells, the ROS levels in BEC^B[a]P^ cells were significantly greater. (Fig. 3D, E). ROS function as crucial intracellular second messengers that play a key role in the initiation and progression of hepatobiliary diseases, including abnormal DR, via the induction of epithelial-mesenchymal transition (EMT) [27]. Compared with BEC^PC^ cells, BEC^B[a]P^ cells markedly increased vimentin expression but decreased E-cadherin expression (Fig. 3F). We further investigated the effects of GRP75 on the B[a]P-induced profibrotic phenotype in BEC^B[a]P^ cells via the knockdown of GRP75 in BEC^B[a]P^ cells (denoted GRP75KD-BEC^B[a]P^). Compared with those in MOCK-BEC^B[a]P^ cells, B[a]P-induced aberrant ER-mitochondrial coupling, mitochondrial Ca^2+^ overload, massive ROS production, and the EMT process were suppressed after GRP75 knockdown (Fig. 3G to L). Moreover, the ability of BEC^B[a]P^ to secrete collagen and activate hepatic stellate cells was markedly inhibited upon GRP75 knockdown (Fig. 3M, N). We also confirmed our data in another cell line, SG231 (supplementary Fig. S4). These results revealed that GRP75 may play important roles in B[a]P-disrupted/induced ER-mitochondrial coupling, mitochondrial Ca^2+^ overload, ROS overproduction, and EMT, promoting a profibrotic phenotype in BEC/SG231^B[a]P^ cells.

### Identification of key monomeric chemicals of TCM that target GRP75

**Fig. 4:**
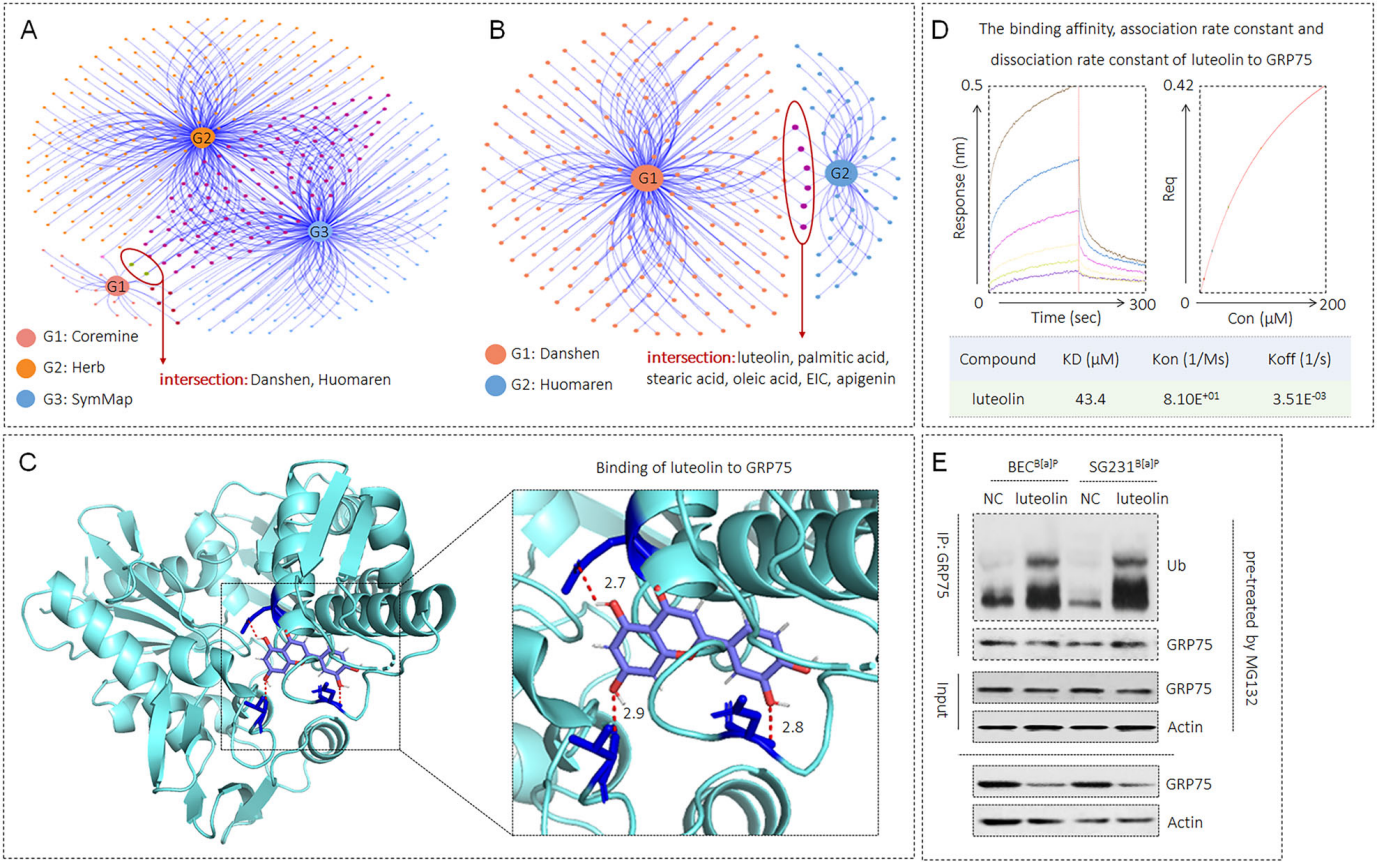
Identification of key monomeric chemicals of TCM that target GRP75. **A** Venn diagram of the Coremine, Herb and SymMap databases, searching for the herbs that exhibit therapeutic effects on cholangitis. **B** Venn diagram of Danshen and Humaren in the TCMSP database, identifying the key monomeric components in these herbs. **C** Molecular docking results of GRP75 and luteolin by PyMol software. **D** Binding of luteolin to recombinant GRP75 protein by a BLI experiment. **E** Co-IP and Western blot analysis of the ubiquitination and expression of GRP75 in both BEC^B[a]P^ and SG231^B[a]P^ cells.

Through mining and analyzing the Coremine, Herb and SymMap databases, we identified two herbs that exhibit therapeutic effects on sclerosing cholangitis: Danshen and Huomaren (Fig. 4A). We subsequently queried the TCMSP database to determine the Chinese herbal monomeric components of these herbs and performed molecular docking simulations with the GRP75 protein. Our docking simulation results revealed that luteolin is the only effective component present in the abovementioned two herbs and can interact effectively with GRP75 (Fig. 4B, C). Therefore, we intend to further investigate the targeted action of luteolin on GRP75. The results of the BLI experiments revealed that luteolin effectively bound to the recombinant GRP75 protein (Fig. 4D). A previous study revealed that luteolin can bind to and subsequently accelerate the ubiquitin-proteasome pathway-mediated degradation of its target protein [28]. In this study, we found that luteolin treatment increased the ubiquitination of GRP75, which in turn decreased the expression of GRP75 in both BEC^B[a]P^ and SG231^B[a]P^ cells (Fig. 4E). Therefore, we hypothesized that the targeted inhibition of GRP75 by luteolin might block B[a]P-induced abnormal DR and hepatic fibrosis.

### Luteolin inhibited B[a]P-induced abnormal DR in vitro

**Fig. 5:**
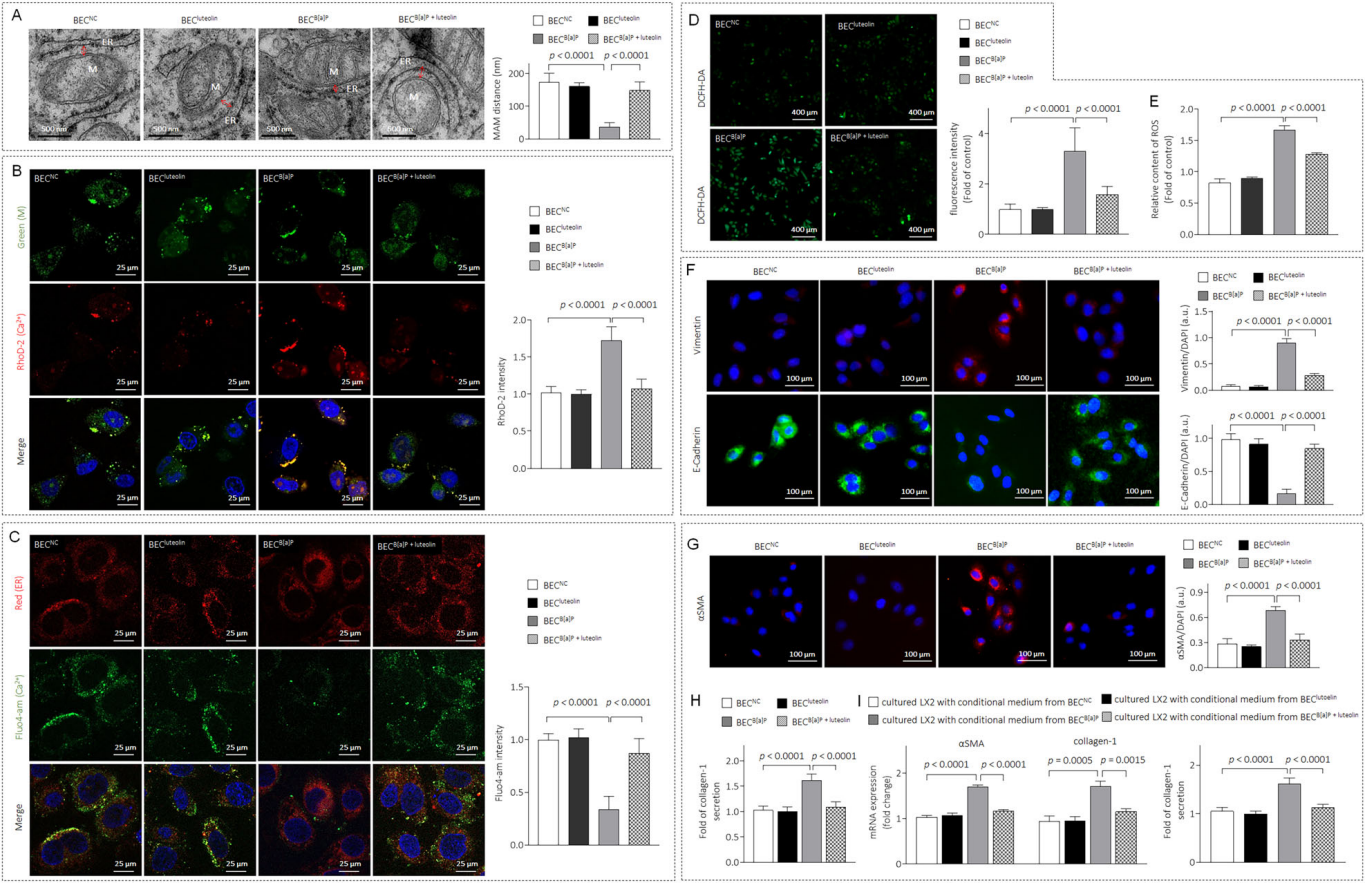
Luteolin inhibited the B[a]P-induced abnormal DR in vitro. BECs cells were treated with 0.0 or 100 nM of B[a]P in the presence or absence of 10 μM of luteolin for 6 culture cycles. **A** TEM image. **B** IF staining analysis of mitochondrial and Ca^2+^ co-localisation. **C** IF staining analysis of ER and Ca^2+^ co-localisation. **D**, **E** Intracellular ROS levels. **F**, **G** IF staining analysis of vimentin, E-Cadherin, and αSMA. **H** Triplicate ELISA analysis of collagen-1. **I** After BECs cells were treated as indicated above, the conditioned media were collected. LX2 cells were treated with such conditioned media, and triplicate qPCR/ELISA analysis of αSMA or collagen-1 was performed. Data were shown as mean ± SD, *n* = 3, and an ANOVA followed by Tukey’s *t*-test was used for between-group comparisons.

We then verified whether luteolin could inhibit B[a]P-induced abnormal DR by constructing an in vitro chronic B[a]P exposure and luteolin intervention model (supplementary Fig. S5). TME analyses indicated that luteolin notably ameliorated B[a]P-induced ER-mitochondrial contact distance shortening (Fig. 5A). Further analysis revealed that luteolin effectively reduced the influx of Ca^2+^ from the ER to mitochondria (Fig. 5B, C), the ROS level (Fig. 5D, E), the EMT (Fig. 5F), and the αSMA and collagen-1 expression and secretion (Fig. 5G, H) induced by chronic B[a]P exposure. Finally, we found that, compared with the conditioned media collected from B[a]P-treated cells, the conditioned media collected from cells treated with B[a]P and luteolin significantly inhibited LX2 activation and collagen secretion (Fig. 5I). These data were confirmed in another cell line, SG231 (Supplementary Fig. S6). These results demonstrate that targeting GRP75 with luteolin inhibits B[a]P-disrupted/induced ER-mitochondrial coupling, mitochondrial Ca^2+^ overload, ROS overproduction, EMT, and the development of a profibrotic phenotype in BEC/SG231^B[a]P^ cells.

### Luteolin inhibited B[a]P-induced abnormal DR in vivo

**Fig. 6:**
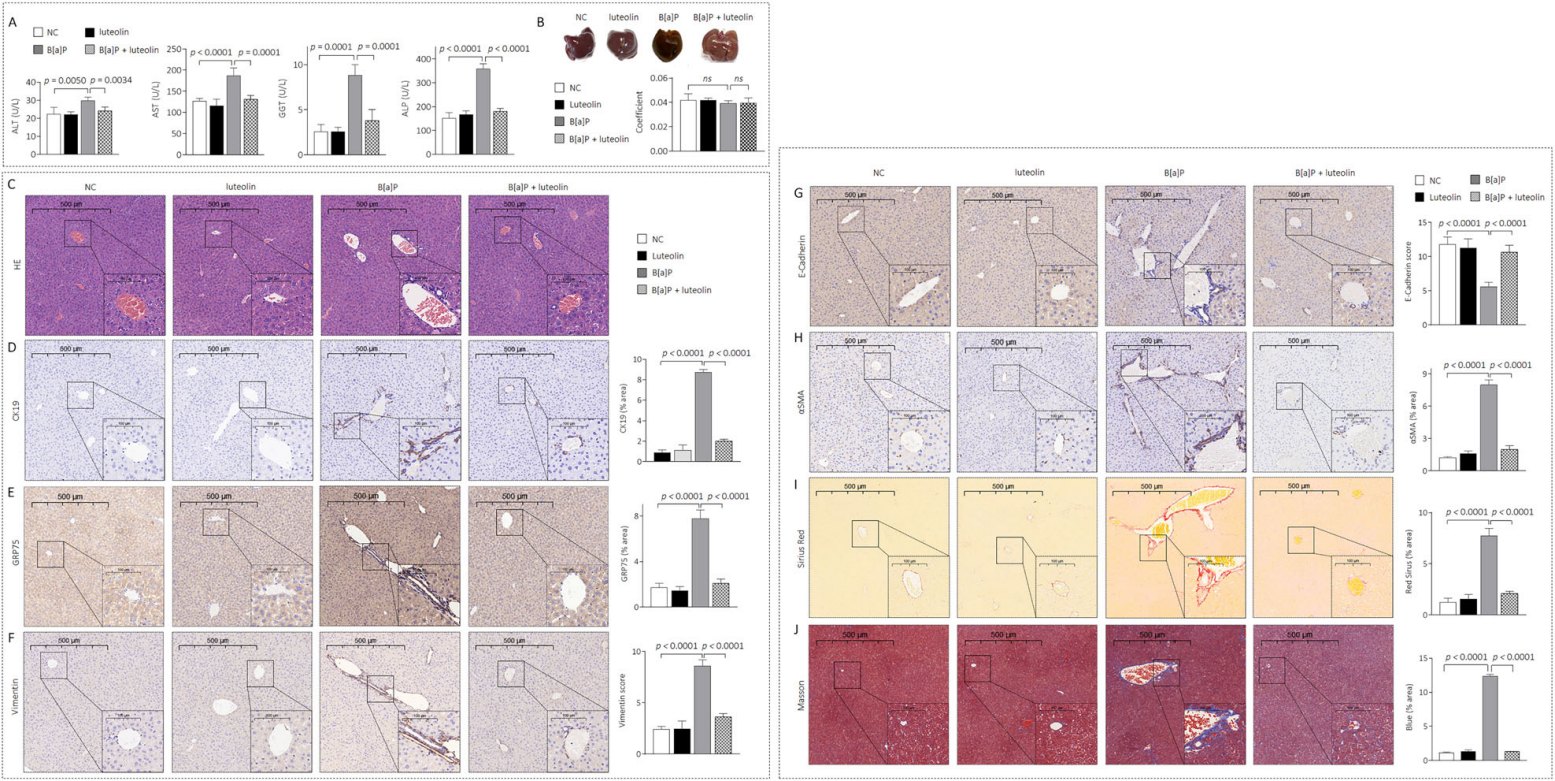
Luteolin inhibited the B[a]P-induced abnormal DR in vivo. **A** Biochemical analysis of blood in NC, luteolin, B[a]P and B[a]P + luteolin groups. **B** Liver gross appearance and coefficient (liver weight/body weight ratio). **C** Liver sections were stained with H&E. **D**–**H** IHC analysis of CK19 (**D**), GRP75 (**E**), Vimentin (**F**), E-Cadherin (**G**), and αSMA (**H**), respectively. **I**, **J** Liver sections were stained with Sirius Red and Masson. Data was shown as mean ± SD, *n* = 5, an ANOVA followed by Tukey’s *t*-test was used for between-group comparisons.

We then sought to determine whether targeted intervention with GRP75 could prevent B[a]P-induced abnormal DR, sclerosing cholangitis-like lesions, and hepatic fibrosis. Mice were randomly divided into 4 groups: NC, luteolin, B[a]P, and a combination of B[a]P and luteolin. Four weeks later, the serum ALT, AST, GGT, and ALP levels were markedly lower in the luteolin combination treatment group than in the B[a]P group (Fig. 6A). There were no notable differences in the gross liver morphology or coefficient among 4 groups (Fig. 6B). Furthermore, compared with those in the B[a]P-treated group, dramatic decreases in the number of reactive bile ducts (Fig. 6C, D), the expression of GRP75 in hepatocytes and BECs (Fig. 6E), the expression of EMT markers, myofibroblast activation and collagen deposition (Fig. 6F–J) were observed in the B[a]P plus luteolin-treated group. These results confirmed that, in vivo, targeting GRP75 with luteolin prevented B[a]P-induced abnormal DR, sclerosing cholangitis-like lesions, and hepatic fibrosis.

### Luteolin reversed B[a]P-induced abnormal DR in vivo

**Fig. 7:**
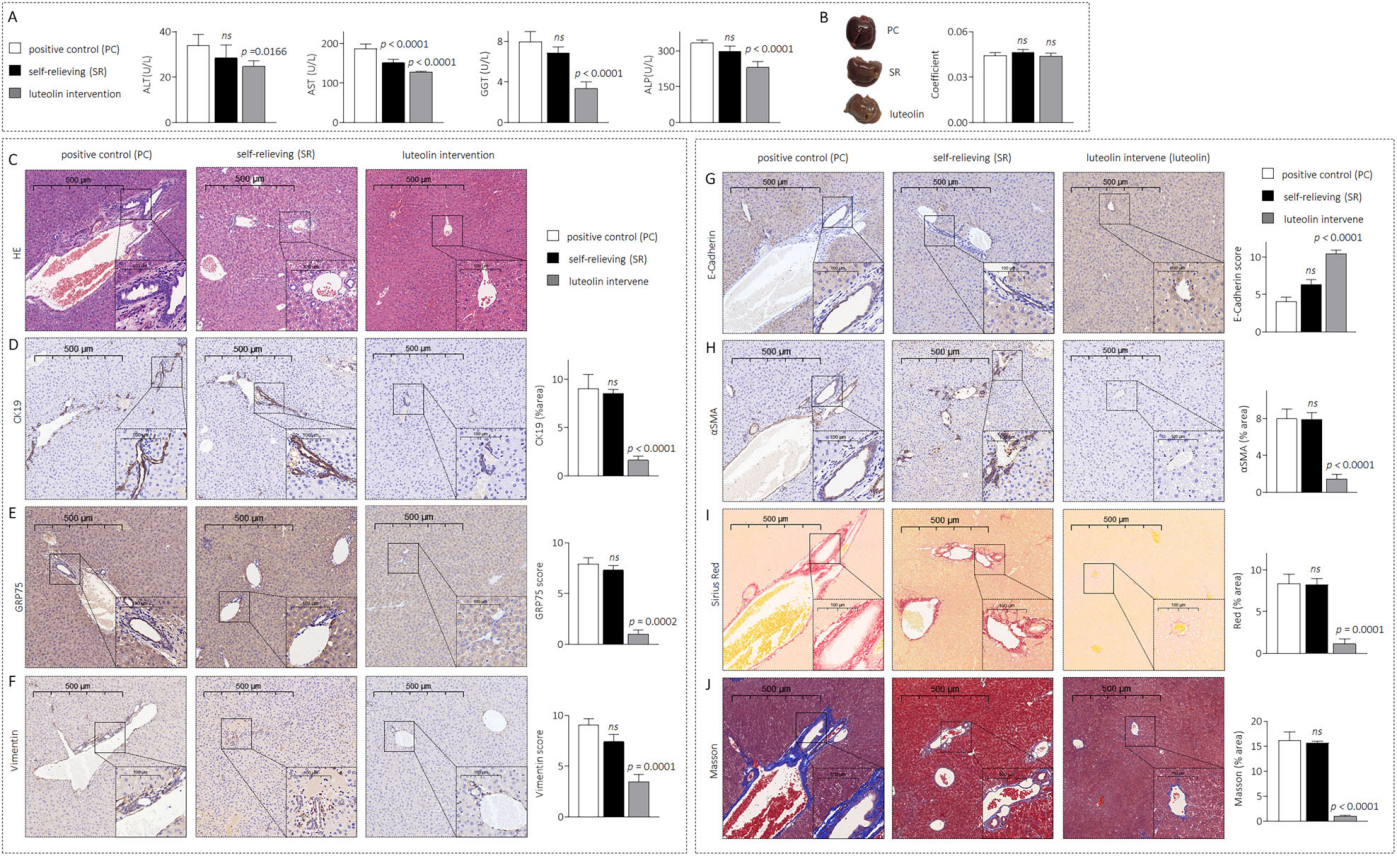
Luteolin reversed the B[a]P-induced abnormal DR in vivo. **A** Biochemical analysis of blood in positive control (PC, mice treated by B[a]P for 4 weeks), self-relieving (removed B[a]P, and continued with conventional feeding for another 2 weeks), and luteolin intervention group (removed B[a]P, and continued to be treated with luteolin for another 2 weeks). **B** Liver gross appearance and coefficient (liver weight/body weight ratio). **C** Liver sections were stained with H&E. **D**–**H** IHC analysis of CK19 (**D**), GRP75 (**E**), Vimentin (**F**), E-Cadherin (**G**), and αSMA (**H**), respectively. **I**, **J** Liver sections were stained with Sirius Red and Masson. Data was shown as mean ± SD, *n* = 5, and an ANOVA followed by Tukey’s *t*-test was used for between-group comparisons.

Finally, we reestablished a model by exposing C57BL/6J model mice to B[a]P for 4 weeks (*n* = 15). After 4 weeks, 10 of these mice were randomly divided into two groups: the self-relieving group (B[a]P was removed, and conventional feeding was continued for another 2 weeks) and the intervention group (B[a]P was removed, and the mice were treated with luteolin for another 2 weeks). There were no significant differences in the serum ALT, GGT, or ALP levels between the positive control (PC) and self-relieving (SR) groups, whereas these levels significantly decreased in the luteolin intervention group (Fig. 7A). No significant differences were observed in gross liver morphology or liver coefficients among these groups (Fig. 7B). Conventional feeding (self-relieving group) slightly decreased bile duct abnormal hyperplasia, EMT markers, αSMA levels, and collagen deposition caused by B[a]P preexposure. However, such reversal effects were significant in the luteolin intervention group (Fig. 7C–J). These findings suggest that even though the mice had undergone early changes indicative of B[a]P exposure-induced chronic liver disease, luteolin could reverse abnormal DR, sclerosing cholangitis-like lesions, and hepatic fibrosis.

## Discussion

Genetic toxicity and epigenetic regulation play important roles in the harmful effects of B[a]P. In fact, the toxicity of B[a]P in target organs depends on the dose, duration of action, and route of administration. On the basis of our previous study, in which the daily intake of B[a]P in the nonoccupationally exposed population was extrapolated to the dose in mice via gavage, we applied 12.5 mg/kg B[a]P to the mice [20]. According to the Global Burden of Disease (GBD) database, in the past 10 years, the prevalence and incidence of cirrhosis in males were significantly higher than those in females (supplementary Fig. S7). Therefore, we selected a dose of 12.5 mg/kg B[a]P as a long-term exposure regimen for male mice to establish a DR model and used 100 nM B[a]P as a long-term exposure regimen for BECs.

DR is found in various acute and chronic liver injuries and plays a key role in liver regeneration and repair, the development of hepatic fibrosis, the development of cirrhosis, and the occurrence and progression of HCC/CCA [29]. DR is a histological feature characterized by abnormal hyperplasia of reactive bile ducts. DR can be classified into three types according to organizational morphology: typical proliferation, atypical proliferation, and early cancer bile duct proliferation [30]. Reactive ductlike cells are the constituent cells of reactive bile ducts; if the damage persists or recurs, they may proliferate abnormally, alter their phenotype, and secrete proinflammatory and profibrotic factors. This process disrupts the liver microenvironment, leading to liver inflammation and fibrosis [31]. This study revealed that B[a]P induces BECs/SG231 cells to acquire the functional properties of mesenchymal cells and produce a profibrotic microenvironment.

Environmental exposure plays a significant role in inducing epigenetic changes, as environmental pollutants can cause nonheritable toxicity by affecting normal gene expression and disrupting cellular functions through epigenetic effects [32]. One of the key mechanisms in epigenetics is DNA methylation [33, 34]. Epidemiological studies have shown that B[a]P causes DNA hypomethylation and the overexpression of inflammatory genes, leading to the development and progression of inflammatory diseases [35]. Previous studies have indicated that B[a]P treatment decreases the expression levels of DNA methyltransferase 1 (DNMT1) and DNMT3A to a certain extent, subsequently decreasing the promoter methylation level and inducing the demethylation process [36, 37]. In this study, low-dose B[a]P exposure continuously activated GRP75 via the epigenetic accumulation of DNA demethylation. Further studies should investigate the potential key methyltransferase subtypes involved in this modification process.

A major mechanism by which B[a]P contributes to disease development is the induction of excess ROS production [38, 39]. Under normal physiological conditions, ROS levels in organisms are maintained in a state of dynamic equilibrium; however, when ROS are overproduced, the integrity and survival of cells are severely threatened, ultimately leading to cell death [40, 41]. Low concentrations of ROS act as second messengers and play crucial roles in cellular signalling [42]. We found that Ca^2+^ overload in BECs led to a moderate increase in ROS, which was maintained at a certain level through long-term exposure to low-dose B[a]P. This moderate increase in ROS activated downstream EMT signalling pathways and induced BECs to produce a profibrotic microenvironment. Research has shown that Ca^2+^ can directly promote the formation of ROS by stimulating enzymes that produce ROS, such as glycerophosphate dehydrogenase [43]. Furthermore, mitochondrial Ca^2+^ uptake primarily increases ROS production by inhibiting the nicotinamide adenine dinucleotide (NAD)/silent information regulator 3 (SIRT3)/superoxide dismutase (SOD2) pathway [26]. Notably, ROS can induce EMT in cells through different mechanisms. Research has shown that ROS activate the nuclear factor kappa-B‌ (NF-κB) inflammatory pathway, which further promotes EMT and the development of pulmonary fibrosis [44]. Additionally, ROS further drives the progression of EMT by activating multiple signalling pathways, including the phosphoinositide 3-kinase (PI3K), protein kinase B (AKT), mechanistic target of rapamycin (mTOR), and mitogen-activated protein kinase (MAPK) pathways [45, 46]. Moderate levels of ROS can also promote epithelial-mesenchymal transition in hepatocytes via the transforming growth factor-beta1 (TGF-β1)/snail-1 pathway, thereby participating in the regulation of the fibrogenic process [27]. In our present study, we found that the ROS in BEC cells were moderately elevated and maintained at a certain level by long-term exposure to low-dose B[a]P. This moderate ROS elevation activated downstream EMT signaling pathways and induced abnormal DR.

Having been identified in several types of chronic fibrotic disorders, EMT is a phenomenon where epithelial cells acquire mesenchymal features, thereby contributing to the fibrogenic process [47, 48]. In several cholangiopathies, cholangiocytes lining the reactive ductules lose some epithelial markers and acquire, in turn, several mesenchymal traits [49, 50]. Neoexpression of S100A4, vimentin, matrix metalloproteinase-2 and snail, which are associated with the downregulation of E-cadherin and K19 in the bile ducts, was observed in histological samples from patients with primary biliary cirrhosis, primary sclerosing cholangitis, and biliary atresia [51, 52]. In this study, we found that BECs treated with B[a]P underwent EMT, downregulated E-cadherin expression, and upregulated vimentin and αSMA expression.

The stress chaperone GRP75 is an essential protein whose overexpression can lead to the development of a variety of diseases and is associated with the development and poor prognosis of CCA [16]. In addition to the already recognized role of GRP75 in activating proliferative and antiapoptotic effects during carcinogenesis, it also plays a key role in EMT through the activation of multiple proteins and pathways [53]. For example, GRP75 activates the PI3K/AKT pathway, inducing EMT to promote the invasion and metastasis of breast cancer cells [54]. GRP75 can also induce EMT in intrahepatic cholangiocarcinoma (ICC) cells and is involved in promoting cell proliferation [55]. We found that elevated GRP75 expression caused ROS overproduction and EMT and induced BECs/SG231 cells to acquire a profibrotic phenotype and produce a profibrotic microenvironment. These findings underscore the potential of GRP75 as a therapeutic target and provide a theoretical foundation for the development of novel therapeutic strategies targeting DR and even ICC.

Luteolin is a pleiotropic flavonoid phytochemical that is widely found in flowers, herbs, and vegetables [56]. Luteolin can improve chronic inflammatory diseases such as atherosclerosis and has protective effects on antioxidant capacity [57]. Luteolin scavenges free radicals and ROS and inhibits the production and elimination of proinflammatory cytokines and enzymes in the body, thereby reducing oxidative stress and alleviating systemic inflammation [58]. Luteolin provides effective protection and treatment in B[a]P-induced lung carcinogenesis via antioxidant effects [59]. Luteolin inhibited the proliferation of HCC cells mainly through cell cycle arrest and apoptosis by targeting AKT1 and steroid receptor coactivators [60]. Despite the increasing evidence supporting the beneficial effects of luteolin, the mechanisms underlying its impact on DR remain unclear. In this study, luteolin reduced GRP75 expression in both hepatocytes and cholangiocytes, reflecting the broad cellular effects of luteolin treatment. And we innovatively revealed a novel therapeutic effect (anti-pro-fibrosis properties via targeting GRP75) of luteolin in cholangiocytes.

## Conclusions

**Fig. 8:**
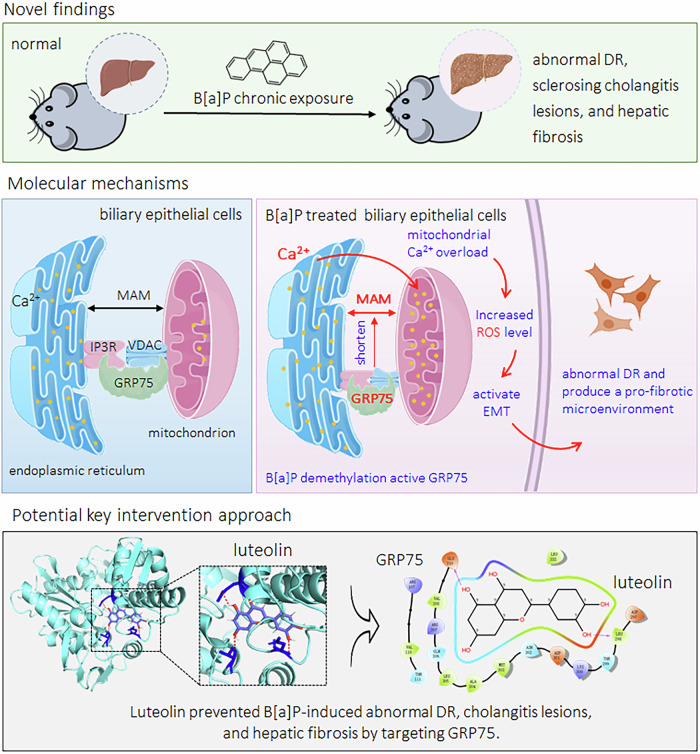
A sketch map summarizing the conclusions, innovations, and potential preventive significance of our present study. The materials for the images (such as mice, liver, protein, endoplasmic reticulum, mitochondrion, cells, etc.) are sourced from BioRender (https://www.biorender.com/).

A low dose of B[a]P induced the overexpression of GRP75, which in turn disrupted ER-mitochondrial coupling, caused mitochondrial Ca^2+^ overload and massive production of ROS, activated EMT, and induced BECs to acquire a profibrotic phenotype and produce a profibrotic microenvironment, ultimately leading to the development of hepatic fibrosis. We also identified a key monomeric chemical of TCM, luteolin, which inhibited the B[a]P-induced abovementioned effects by targeted intervention with the GRP75 protein (Fig. 8).

## Supplementary information

Supplementary Files

Original western blots

## Data Availability

The datasets used and/or analyzed during the current study are available from the corresponding author on reasonable request.
