# Supplementary material for: Mechanisms and targeted prevention of abnormal ductular reaction caused by a low concentration of Benzo(a)pyrene
Source: Cell Death Dis. 2025 Oct 7;16(1):714. doi: 10.1038/s41419-025-08043-8 (PMC12504665; doi:10.1038/s41419-025-08043-8)
Supplement: Supplementary file 1 — Supplementary Files [file 41419_2025_8043_MOESM1_ESM.docx]

**Title**

Mechanisms and Targeted Prevention of Abnormal Ductular Reaction Caused by a Low Concentration of Benzo(a)pyrene

**Author list**

Xinru Du ^1, 2, 3,^ **^*^**, Yancheng Gao ^1, 2, 3,^ **^*^**, Sisi Song ^2, 3^, Qinming Hui ^2, 3^, Zhendong Wang ^2, 3^, Mengyue Ji ^1^, Maoxuan Li ^2, 3^, Shuoke Duan ^2, 3^, Sha Liu ^2,3^ , Ziyi Wang ^2, 3^, Yue Ma ^2, 3^, Ye Yang ^2, 3,^ **^🖂^**, Chunxiao Zhou ^1,^ **^🖂^**, and Yuan Li ^1, 2, 3,^ **^🖂^**

**Authors’ affiliations**

1. Department of Gastroenterology, The Affiliated Suzhou Hospital of Nanjing Medical University, Suzhou 215002, Jiangsu Province, China.
2. The Key Laboratory of Modern Toxicology, Ministry of Education, School of Public Health, Nanjing Medical University, Nanjing 211166, Jiangsu Province, China.
3. School of Public Health, Key Laboratory of Public Health Safety and Emergency Prevention and Control Technology of Higher Education Institutions in Jiangsu Province, Nanjing Medical University, Nanjing 211166, Jiangsu Province, China.

**Equal contributions***

Xinru Du and Yancheng Gao contributed equally to this work.

**Corresponding author informations^🖂^**

To whom correspondence should be addressed at: Dr. Yuan Li, E-mail: liyuan@njmu.edu.cn; or at Dr. Chunxiao Zhou, E-mail: zhouchunxiao@njmu.edu.cn; or at Dr. Ye Yang, Email: [yangye@njmu.edu.cn](mailto:yangye@njmu.edu.cn).

**Running title:** B[a]P-induced abnormal DR via GRP75-regulated Mito-disfunction

**Inventory**

1. Supplementary Materials and Methods, 3 to 8
2. Supplementary Tables, pages 9 to 10
3. Supplementary Figures, pages 11 to 18
4. Supplementary References, pages 19

**1. SUPPLEMENTARY MATERIALS AND METHODS**

**Detection of serum biochemical parameters**

Blood was collected from the abdominal aorta of mice (n = 5), Subsequently, the blood samples were centrifuged at 3000 g for 10 min to collect serum within 1 h and stored at -80℃ for later use. The alanine aminotransferase (ALT), aspartate aminotransferase (AST), gamma glutamyl transpeptidase (GGT), and alkaline phosphatase (ALP) levels were analyzed triplicately using a 7100 automatic biochemical analyser (Hitachi, Japan) as we described previously ^1^.

**Cell transfection**

For cell transfection, the negative control NC-siRNA and GRP75-siRNA were purchased from Generay Biotech (Shanghai, China, details were listed in supplementary Table. S1). Briefly, 0.0 or 20 nM of siRNAs were mixed with lipofectamine 3000 reagent (Invitrogen, Carlsbad, USA) and mediums containing 10% FBS without antibiotics. The experiment was replicated triplicately. After 12 h of transfection, the cells were cultured in fresh mediums supplemented with 10% FBS for another 24 h before being used for other experiments as we described previously ^2^.

**DNA methylation analysis**

The genomic DNA was isolated by TIANamp Genomic DNA Kit (Qiagen, Beijing China, No. DP304). EpiTect Kit (Qiagen) was employed to modify the genomic DNA with sodium bisulfite. DNA methylation was analyzed using SYBR Green-based quantitative methylation specific PCR (qMSP) as described previously ^3^. Primer sequences used listed in supplementary Table. S2. The experiment was replicated triplicately. Briefly, 1 µl of bisulfite-treated DNA template was mixed with 10 µl of 2 × Power SYBR Green Master Mix and a pair of primers at a final concentration of 400 nM. The PCR conditions included initial incubation at 50°C for 2 min, denaturing at 95°C for 10 min, 40 cycles of denaturing at 95°C for 15 s, and annealing at 60°C for 1 min. The percentages of methylation in the sample were estimated by the formula: methylation (%) = [ M/(M + U)] × 100% = [1/(1 + U/M)] × 100% = [1/(1 + 2^ΔCt^))] × 100%. Annotation: M: methylated; U: unmethylated.

**Fluorescence mitochondrial calcium and ER calcium imaging**

The experiment was replicated triplicately. For mitochondrial and calcium imaging, cells were seeded into confocal dishes. The cells were pre-incubated with 20 nM Mito-Tracker Green (Beyotime) and 4 µM Rhod-2 (Yeasen Co. Ltd, Shanghai, China) in a dark chamber at 37°C for 20 min. For ER calcium, the cells were pre-incubated with 20 nM ER-Tracker Green (Beyotime) and 4 µM Fluo4- am (Thermo Fisher, Shanghai, China) in a dark chamber at 37°C for 20 min. The specimens were observed and imaged by a LSM900 with airyscan-2 confocal fluorescence microscope (Carl Zeiss, Oberkochen, Germany). Intensity in the regions of interest was quantified using Image J software (NIH freeware).

**Transmission electron microscopy (TEM)**

The experiment was replicated triplicately. Cells were seeded into T25 culture flasks (Thermo Fisher), cultured, and collected at 80% fusion and immediately fixed in 4% glutaraldehyde (Leagene, Beijing, China) at 4°C for 6 h. Ultrathin sections (60 nm) were cut using an ultramicrotome (Leica EM FC7, Wetzlar, Germany), collected onto 200-mesh copper grids, and counterstained with uranyl acetate and lead citrate to enhance contrast. Sections were examined using a TEM (JEOL JEM-1400plus, Tokyo, Japan) operating at 120 kV.

**Reactive Oxygen Species (ROS) Staining and quantification**

The ROS assay kit (Beyotime) was used according to the manufacturer’s instructions. The experiment was replicated triplicately. Cells were cultured in a 6-well plate, treated with B[a]P stimulation, and then incubated with an appropriate amount of diluted 2',7'-Dichlorodihydrofluorescein diacetate (DCFH-DA) for 20 min at 37℃, followed by washed three times with serum-free medium. The fluorescent signal was observed using a fluorescence microscope (Axio Vert. AE, Zeiss, Germany), and DCFH-DA fluorescence intensity was measured via a multi-well plate reader (Bio-Rad, Shanghai, China) at Ex (λ) 488 nm and Em (λ) 525 nm as we described previously ^4^.

**Quantitative real-time polymerase chain reaction (qRT-PCR)**

Total RNA was isolated using TRIzol reagent (Thermo Fisher) and reverse transcribed into cDNA using AMV Reverse Transcriptase (Promega, Madison, USA). qRT-PCR was performed in triplicate using a Light Cycler 96 machine (Roche Applied Science, Basel, Switzerland) with SYBR Green Master Mix (Vazyme Biotech, Nanjing, China). Primers used were listed in supplementary Table. S2. The β-actin was amplified to ensure cDNA integrity and to normalize expression. Fold changes in expression of each gene were calculated by a comparative threshold cycle (Ct) method using the formula 2^-(ΔΔCt)^ as we described previously ^5^.

**Western blot**

Tissues/cells were lysed with cold RIPA lysis buffer (Beyotime) and the protein concentrations were measured with the bicinchoninic acid assay Kit (BCA, Beyotime). The experiment was replicated triplicately. Samples containing equal amounts of protein were loaded onto sodium dodecyl sulfate - polyacrylamide gel electrophoresis for electrophoresis and subsequently transferred onto a polyvinylidene fluoride membrane. After blocking with tris-buffered saline buffer (TBS, all the above reagents from Beyotime), the membrane was incubated with the antibody listed in supplementary Table. S3. The protein levels were detected and visualized using an ultra-sensitive enhanced chemiluminescent (ECL) substrate as we described previously ^1^.

**Immunofluorescence staining (IF)**

The *in vitro* experiment was replicated triplicately. Cells were fixed with 4% paraformaldehyde, permeabilized with 0.1% Triton X-100, and blocked with blocking buffer for immunol staining for 1 h. Subsequently, the cells were incubated with primary antibodies overnight at 4°C, as the antibodies used were listed in supplementary Table. S3, followed by incubation with secondary antibodies for 1 h at room temperature in the dark. After washing, cells were stained with 4′, 6-diamidino-2-phenylindole (DAPI, all the above reagents from Beyotime) and analyzed under a fluorescent microscope (Zeiss).

Immunofluorescence Co-staining of CK19 and GRP75 in paraffin-embedded liver tissue (n = 5). Formalin-fixed, paraffin-embedded liver sections underwent dewaxing, rehydration, and heat-induced antigen retrieval in citrate buffer. After blocking with 5% normal donkey serum, sequential co-staining was performed: sections were first incubated with mouse anti-CK19 overnight at 4°C, labeled with Alexa Fluor 488-conjugated secondary antibody for 2 h at room temperature, followed by rabbit anti-GRP75 overnight and Alexa Fluor 594-conjugated secondary antibody. Nuclei were counterstained with DAPI. Images were captured and analyzed using ZEN software (Zeiss), with fluorescence intensity quantified across multiple fields to evaluate uniformity and specificity of staining as we described previously ^6^.

**Histological analyses**

Liver tissues (n = 5) were formalin-fixed, paraffin-embedded, and 6 μm sections were prepared and stained with hematoxylin and eosin (H&E), Sirius Red and Masson’s trichrome stain to assess the degree of hepatic fibrosis. The images were captured using a panoramic-scan digital slice scanning system (3DHISTECH Co. Ltd, Budapest, Hungary). For the quantification of Sirius Red and Masson, photomicrographs from 5 random fields of view using a 10 × objective were taken from each section, and the ratio of red/blue collagen areas to the total area was measured using ImageJ software as we describe previously ^1^.

**Immunohistochemistry (IHC)**

Sections (n = 5) mounted on silanized slides were dewaxed in xylene; dehydrated in ethanol; boiled in 0.01 M citrate buffer (pH 6.0) for 20 min in a microwave oven; and then incubated with 3% hydrogen peroxide for 5 min. Then sections were incubated in 10% normal bovine serum albumin for 5 min, followed by incubation with primary antibody at 4˚C overnight. The antibodies used were listed in supplementary Table. S3. The slides were then incubated with a horseradish peroxidase-conjugated at room temperature for another 30 min. Samples were then visualized using DAPI, dehydrated, cleared, mounted, and photographed under a panoramic-scan digital slice scanning system (3DHISTECH). The quantitation of immunostaining was performed by two independent researchers who were blinded regarding the animal group details. The score of GRP75 was semi-quantified by quick-score (Q-score) based on intensity and heterogeneity as we describe previously ^4^. For the quantification of CK19 and α-SMA, photomicrographs from 5 random fields of view using 10 × objective were taken from each section, the ratio of positive area to the total area were measured using ImageJ software as we describe previously ^1^.

**Enzyme-linked immunosorbent assay (ELISA)**

The mouse collagen-1 Elisa Kit was purchased from Enzyme-linked Biotechnology Co., Ltd. (Shanghai, China). The experiment was replicated triplicately. For collagen-1 detection, we add 100 μl of standard, control, or sample per well. Each well was aspirated and washed with a wash buffer (400 μl); this process was repeated three times. Subsequently, the buffer was removed. The plate was then inverted and blotted against clean paper towels. Next, 100 μl of the substrate solution was added to each well and incubated for 20 min. The optical density of each well was determined within 30 min using a microplate reader at 450 nm as we described previously ^1^.

**Data mining and bioinformatics analysis**

The data on the mutation, promoter methylation and expression levels of genes were obtained from cBio Cancer Genomics Portal (http://www.cbioportal.org/) and UCSC Xena (https://xena.ucsc.edu/). The data were all obtained from The Cancer Genome Atlas (TCGA). The sketch maps were created with BioRender.com.

**2. SUPPLEMENTARY TABLES**

**Table. S1. siRNAs used in this study**

| siRNAs | Web Link | Used |
| --- | --- | --- |
| NC | https://datasheets.scbt.com/sc-37007.pdf | 20 nM |
| GRP75 | https://datasheets.scbt.com/sc-35520.pdf | 20 nM |

**Table. S2. Primers used in this study**

| mRNAs | primers |
| --- | --- |
| *GRP75* | F: 5’- TGGGATTGTGCACGTTTCTG -3’  R: 5’- TGGGATTGTGCACGTTTCTG -3’ |
| *α-SMA* | F: 5’- GTTCAGTGGTGCCTCTGTCA -3’  R: 5’- ACTGGGACGACATGGAAAAG -3’ |
| *Collagen-1* | F: 5’- GCTCCTCTTAGGGGCCACT -3’  R: 5’- CCACGTCTCACCATTGGGG -3’ |
| *β-actin* | F: 5’- GACCTGACCTGCCGTCTA -3’  R: 5’- GGAGTGGGTGTCGCTGT -3’ |
| *GRP75-methylated* | F: 5’- GCTGGTCTCGAACTCCTG -3’  R: 5’- GCTCATGCCTGTAATCCC -3’ |
| *GRP75-unmethylated* | F: 5’- ATTTCGTGATTAGTCGTT -3’  R: 5’- CATACCTATAATCCCAAC -3’ |

**Table. S3. Antibodies used for IF, Western blot or IHC**

| Antibodies | Source | Product No. | Dilution |
| --- | --- | --- | --- |
| GRP75 | Abcam | ab2799 | 1: 100 (IF)  1: 1000 (WB)  1: 100 (Co-IP)  1: 100 (IHC) |
| CK19 | Thermo Fisher | MA5-12319 | 1: 50 (IF)  1: 100 (IHC) |
| αSMA | Cell Signaling Technology | 19245 | 1: 50 (IF)  1: 100 (IHC) |
| vimentin | Cell Signaling Technology | 5741 | 1: 50 (IF)  1: 100 (IHC) |
| E-cadherin | Cell Signaling Technology | 3195 | 1: 50 (IF)  1: 100 (IHC) |
| Alexa Fluor 488 | Thermo Fisher | AS594HRP | 1: 500 (IF) |
| Alexa Fluor 594 | Thermo Fisher | AS488HRP | 1: 500 (IF) |
| ubiquitin | MedChemExpress | YA022 | 1: 500 (WB) |
| Actin | Cell Signaling Technology | 4970 | 1: 1000 (WB) |

**3. SUPPLEMENTARY FIGURES**

**Fig. S1. Effects of B[a]P chronic exposure on DR in SG231 cells.**


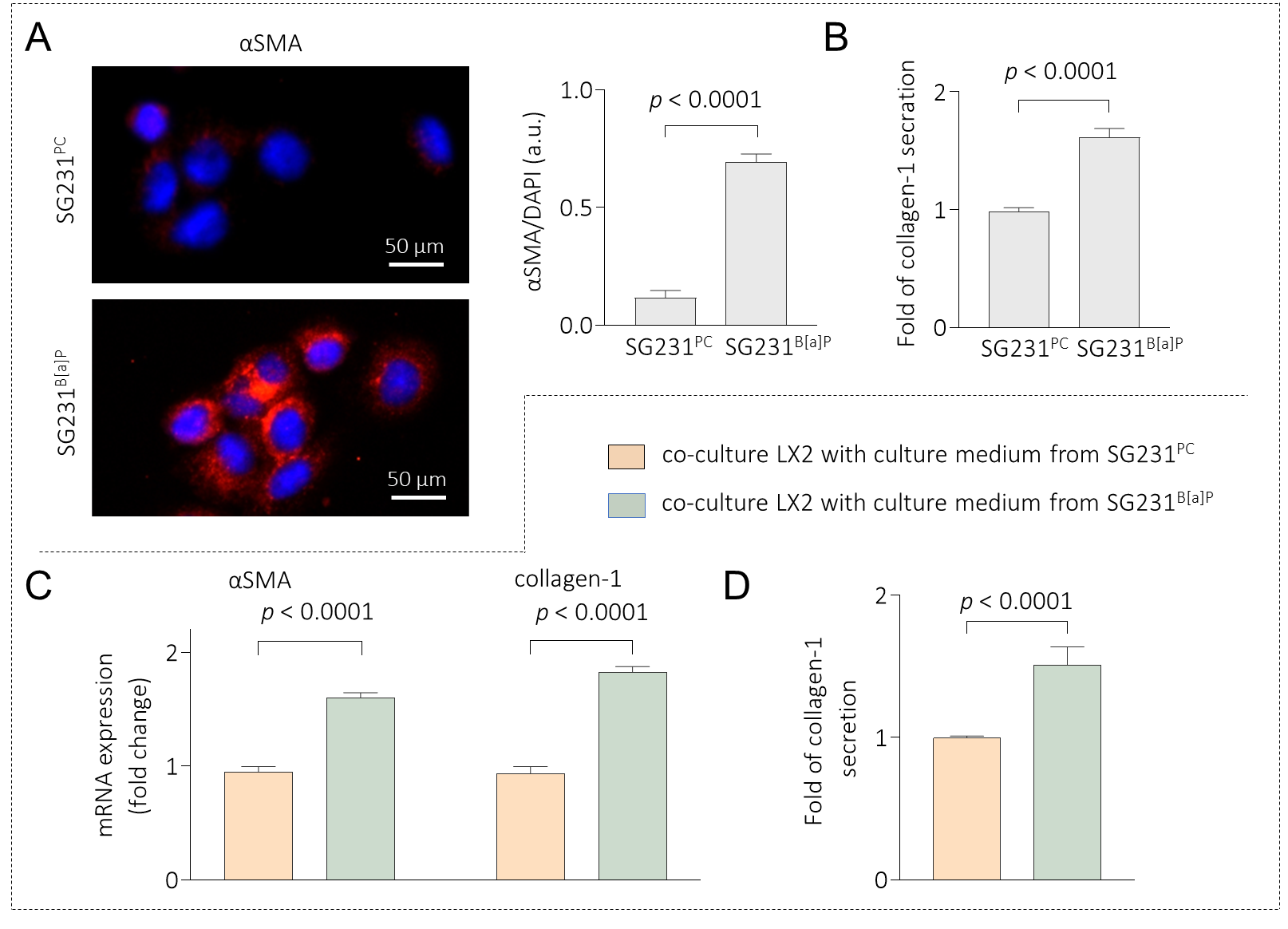


(A) IF staining analysis of αSMA, and (B) ELISA analysis of collagen-1 in SG231^PC^ and SG231^B[a]P^ cells. (C) Triplicate qPCR, and (D) ELISA analysis of αSMA and/or collagen-1 expression/secretion in LX2 cells treated by conditional mediums. Data was shown as mean ± SD, n =3, a two-tailed Student's t-test was used for between two group comparison.

**Fig. S2. Effects of B[a]P chronic exposure on expression/promoter methylation of GRP75 in SG231 cells.**


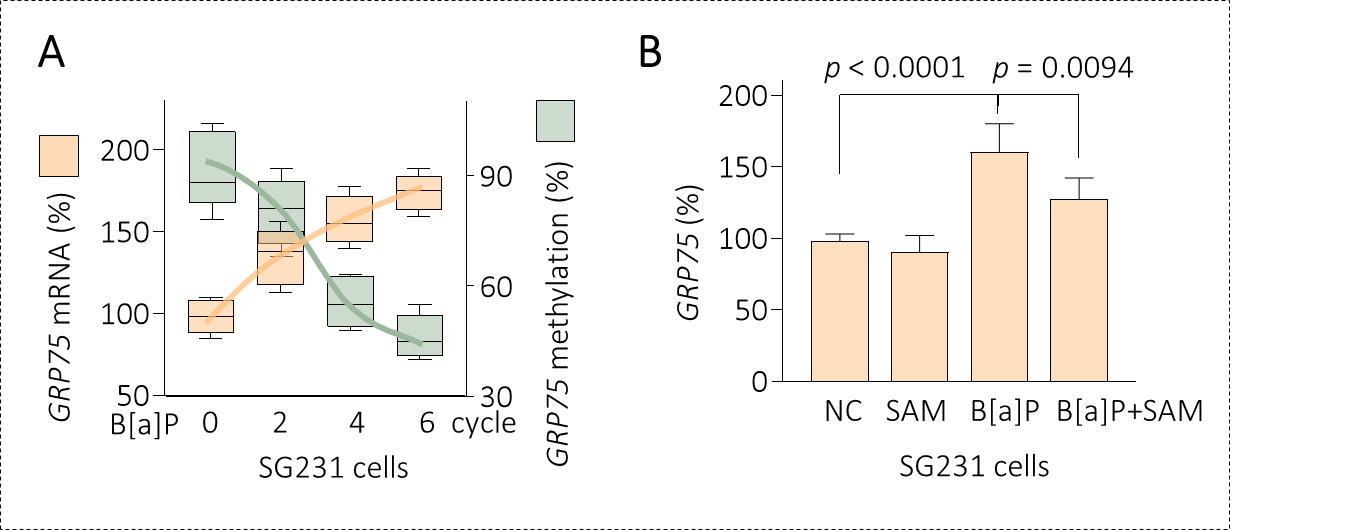


Triplicate qMSP and qPCR analysis of *GRP75* expression and promoter methylation in SG231 cells after the treatment as indicated. Data was shown as mean ± SD, n =3, an ANOVA followed by Tukey’s t test was used for between-group comparisons.

**Fig. S3. The co-localization of GRP75 and cholangiocytes marker CK19.**


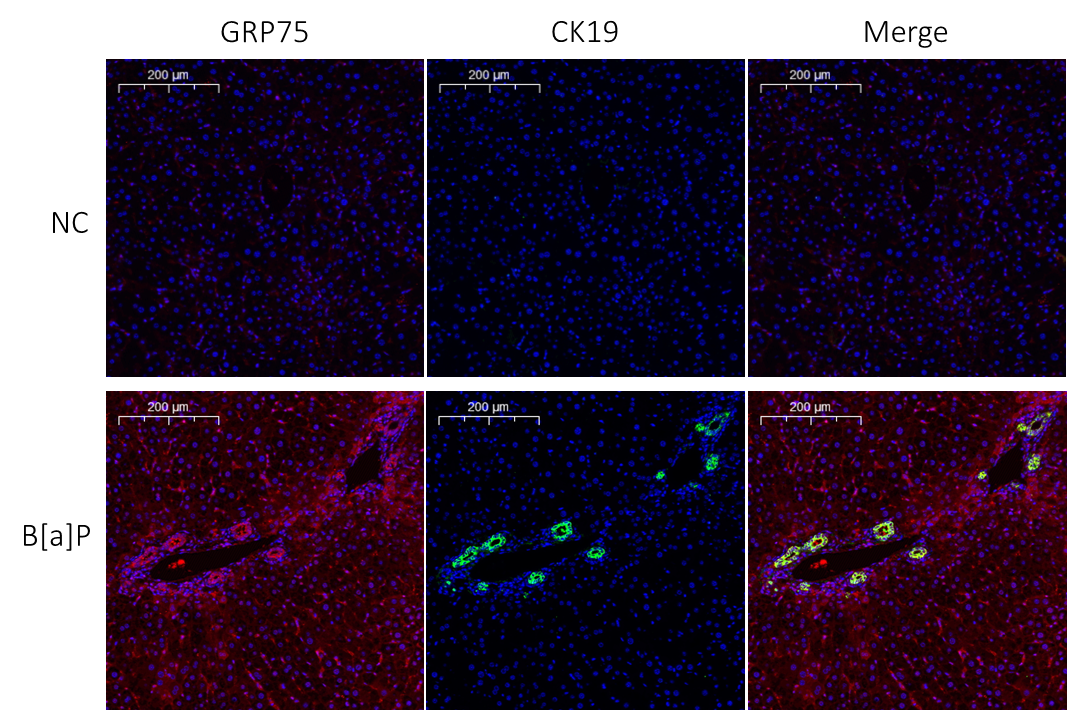


The co-localization of GRP75 and cholangiocytes marker CK19 (red represents GRP75, green represents CK19).

**Fig. S4. B[a]P causes abnormal ER-mitochondrial coupling, mitochondrial Ca^2+^ overload, excessive ROS production, and epithelial-to-mesenchymal transition (EMT) via GRP75 in SG231 cells.**

**
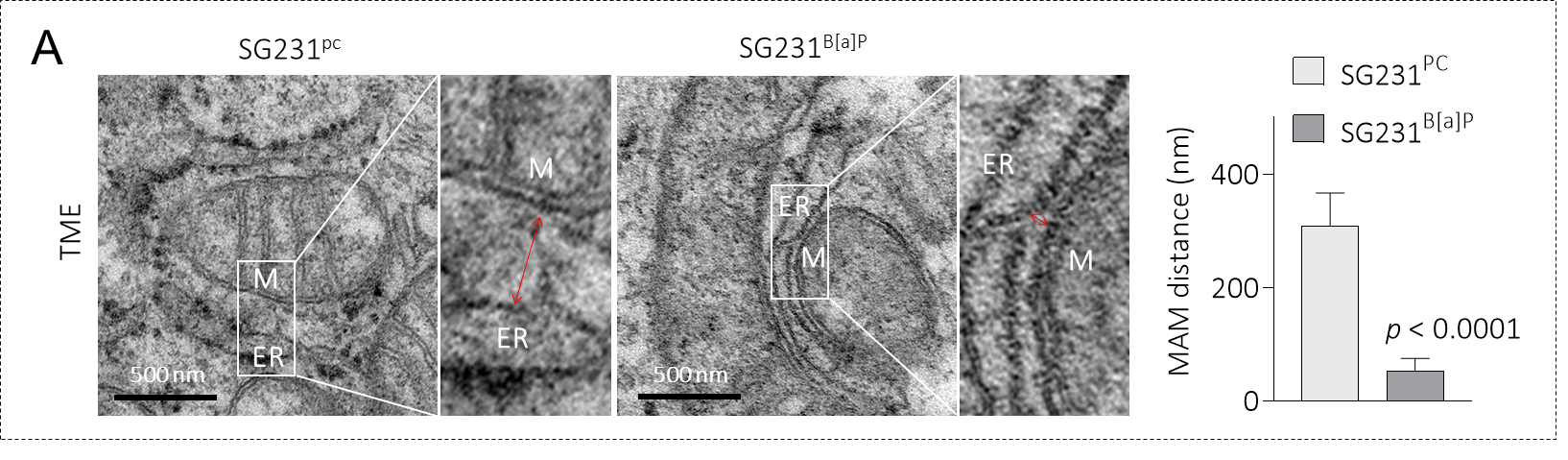
**


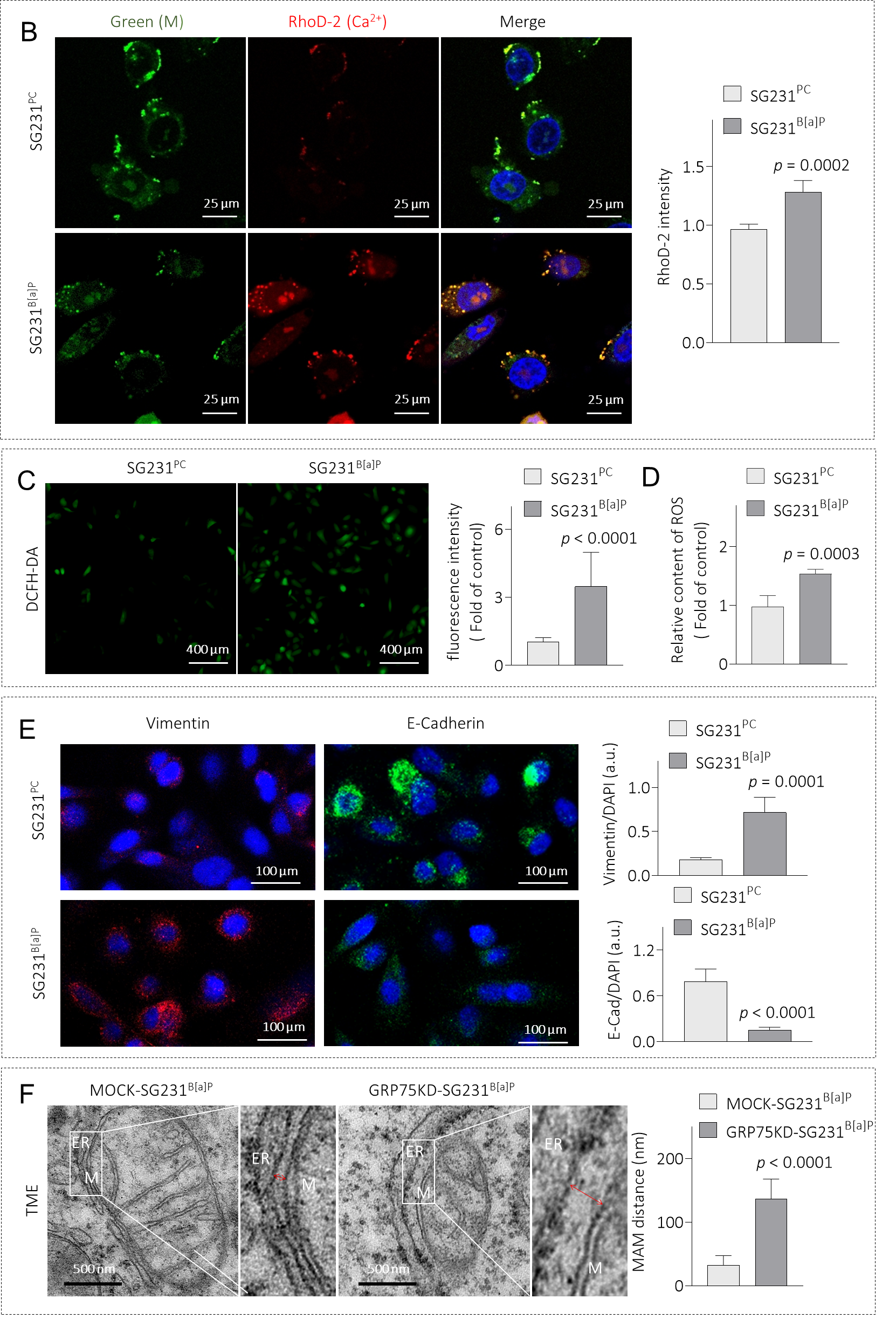


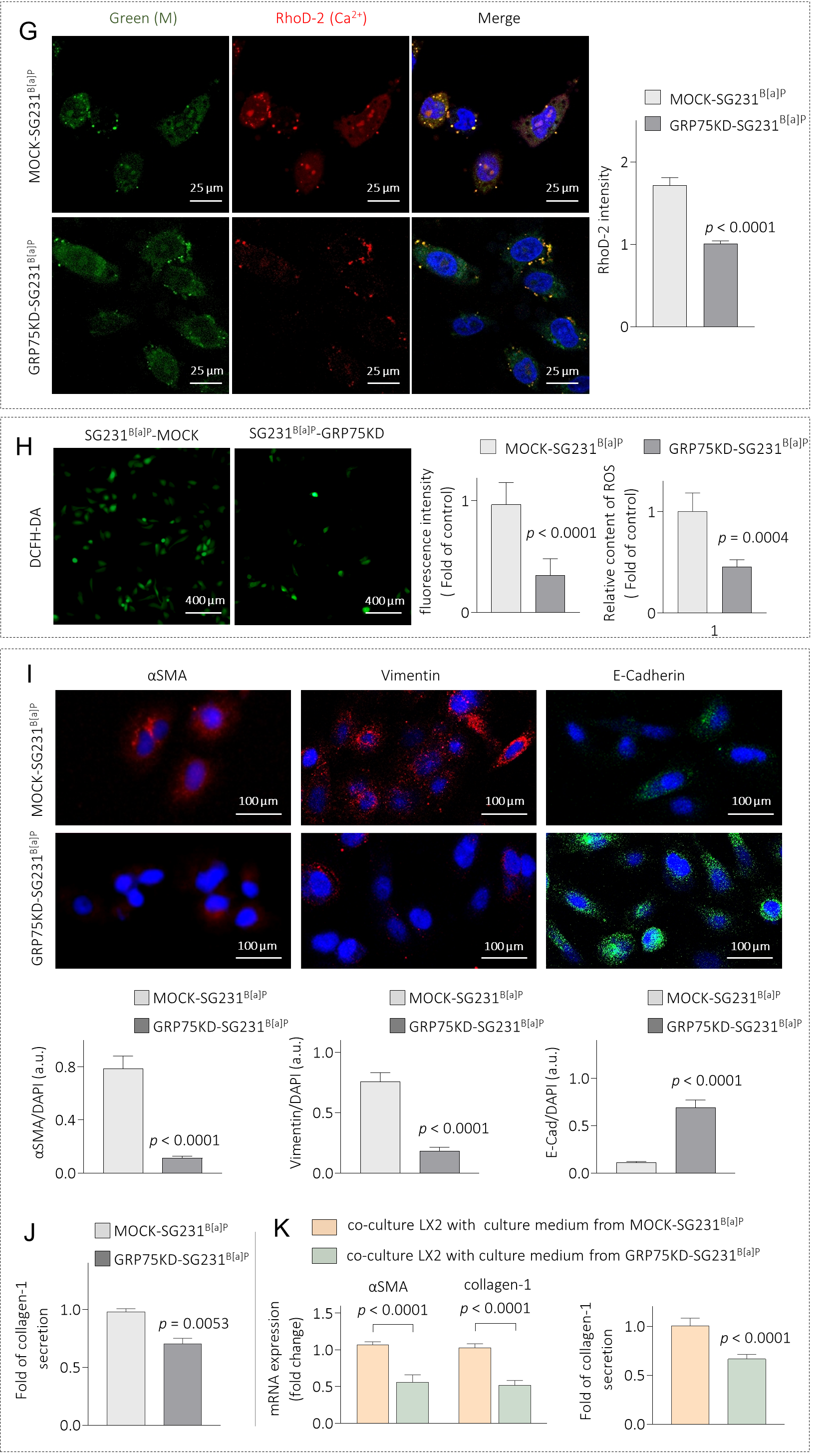


(A to E) In SG231^PC^ and SG231^B[a]P^ cells. (A) TEM images. (B) IF staining analysis of mitochondrial and Ca^2+^ co-localisation. (C and D) Intracellular ROS levels by DCFH-DA fluorescence and multi-well plate reader detection. (E) IF staining analysis of vimentin and E-Cadherin. (F to J) SG231^B[a]P^ cells were transfected by NC- or GRP75-siRNA. (F) TEM images. (G) IF staining analysis of mitochondrial and Ca^2+^ co-localisation. (H) Intracellular ROS levels. (I) IF staining analysis of vimentin, E-Cadherin, and αSMA. (J) Triplicate ELISA analysis of collagen-1. (K) After LX2 cells were treated by conditional mediums collected from MOCK-SG231^B[a]P^ or GRP75-KD-SG231^B[a]P^ cells, triplicate qPCR/ELISA analysis of αSMA or collagen-1 were performed. Data was shown as mean ± SD, n =3, a two-tailed Student's t-test was used for between two group comparison.

**Fig. S5. Constructing an *in vitro* B[a]P chronic exposure and luteolin intervention model in BEC cells.**


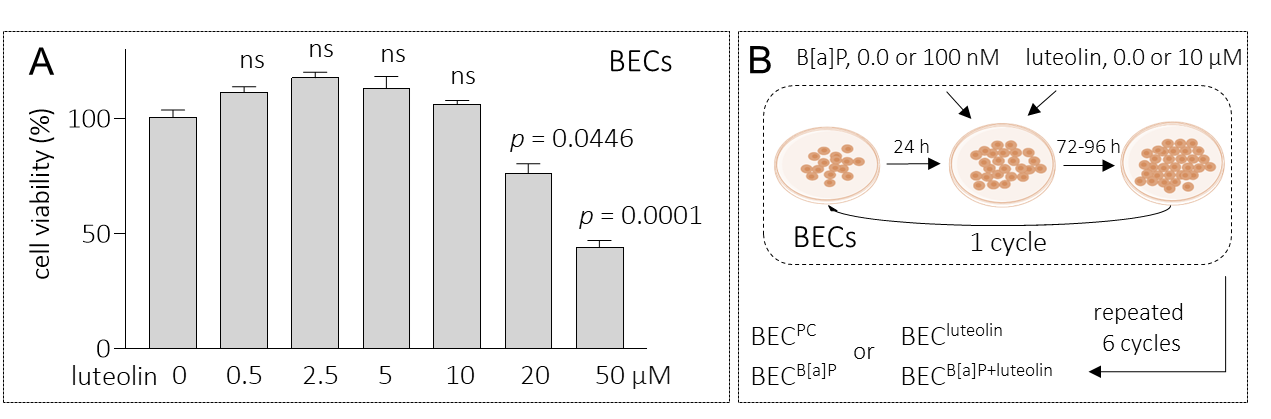


(A) Dosage selection basis of luteolin in this study. (B) A sketch Map for constructing an *in vitro* B[a]P chronic exposure and luteolin intervention model in BEC cells. Data was shown as mean ± SD, n =3, an ANOVA followed by Tukey’s t test was used for between-group comparisons.

**Fig. S6.** **Luteolin inhibited the B[a]P-induced DR in SG231 cells.**

**
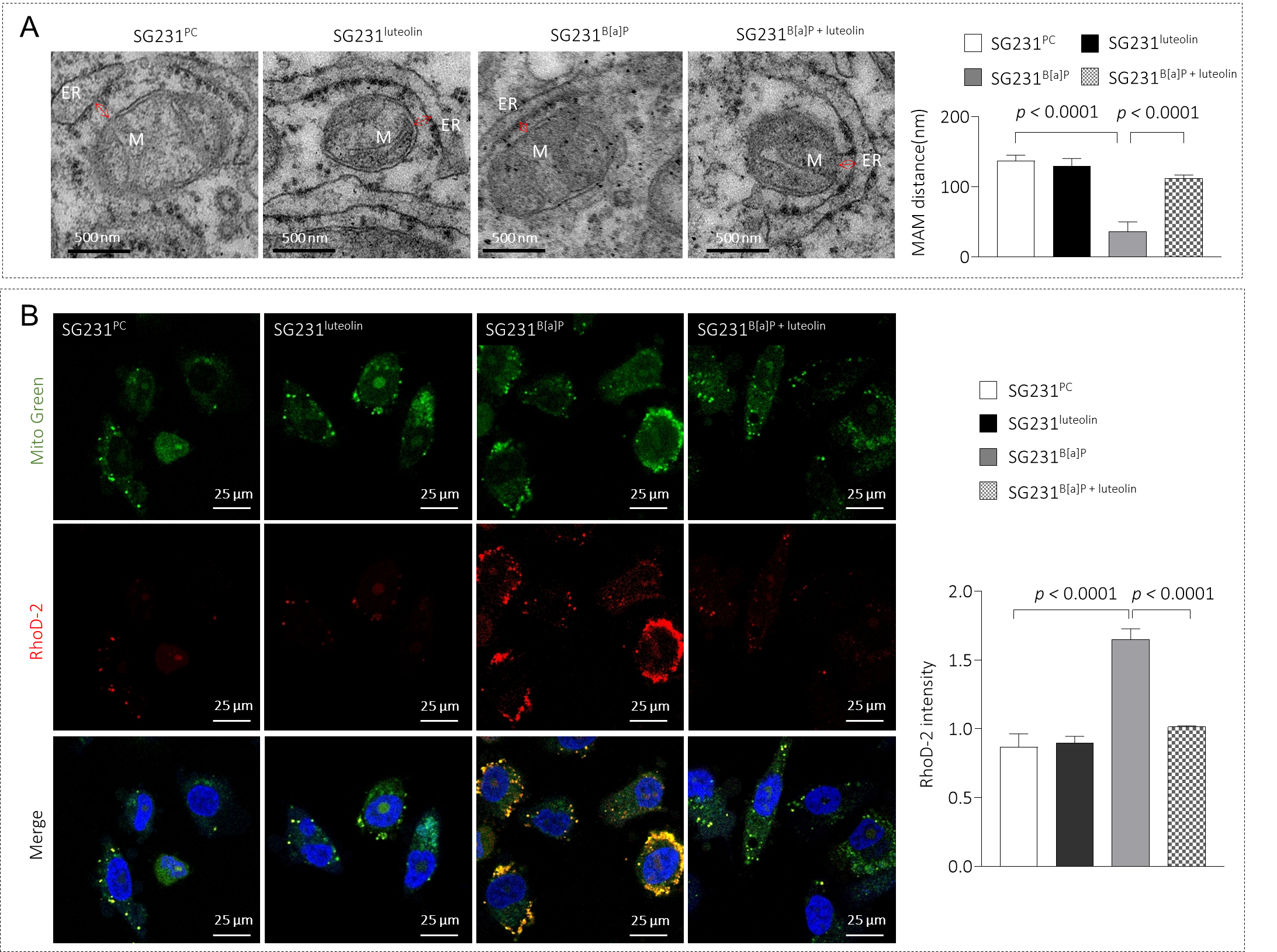
**


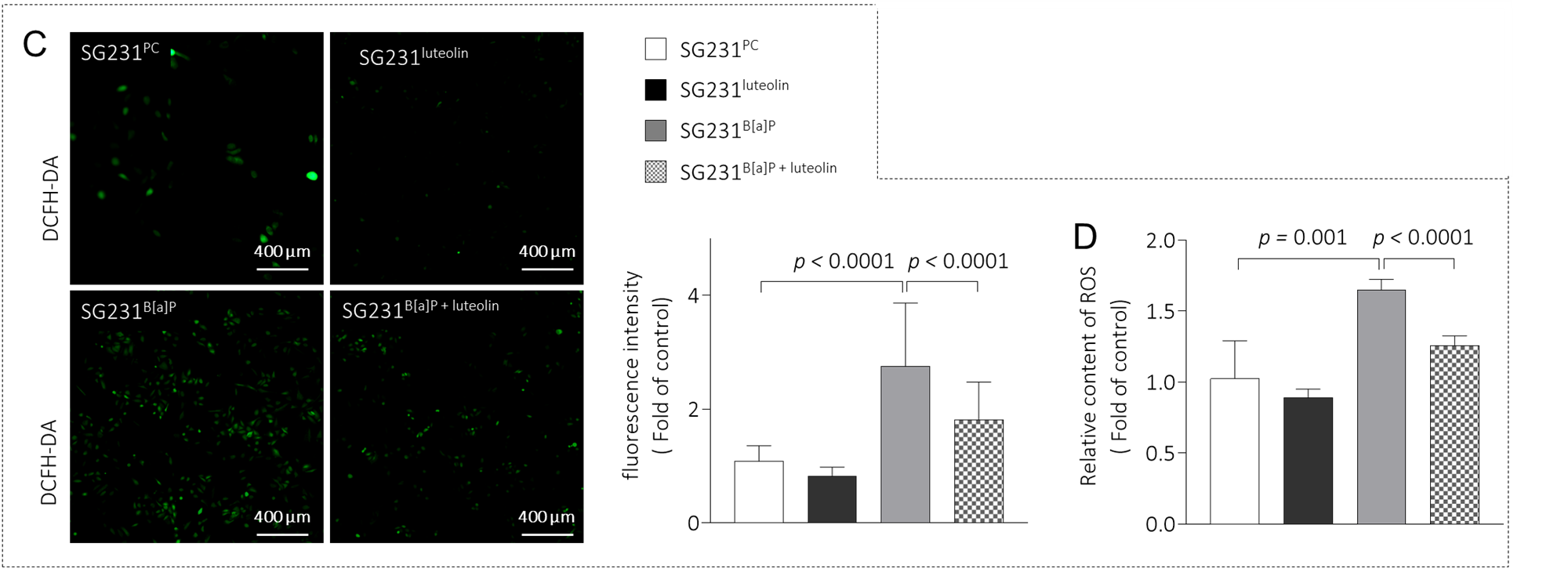


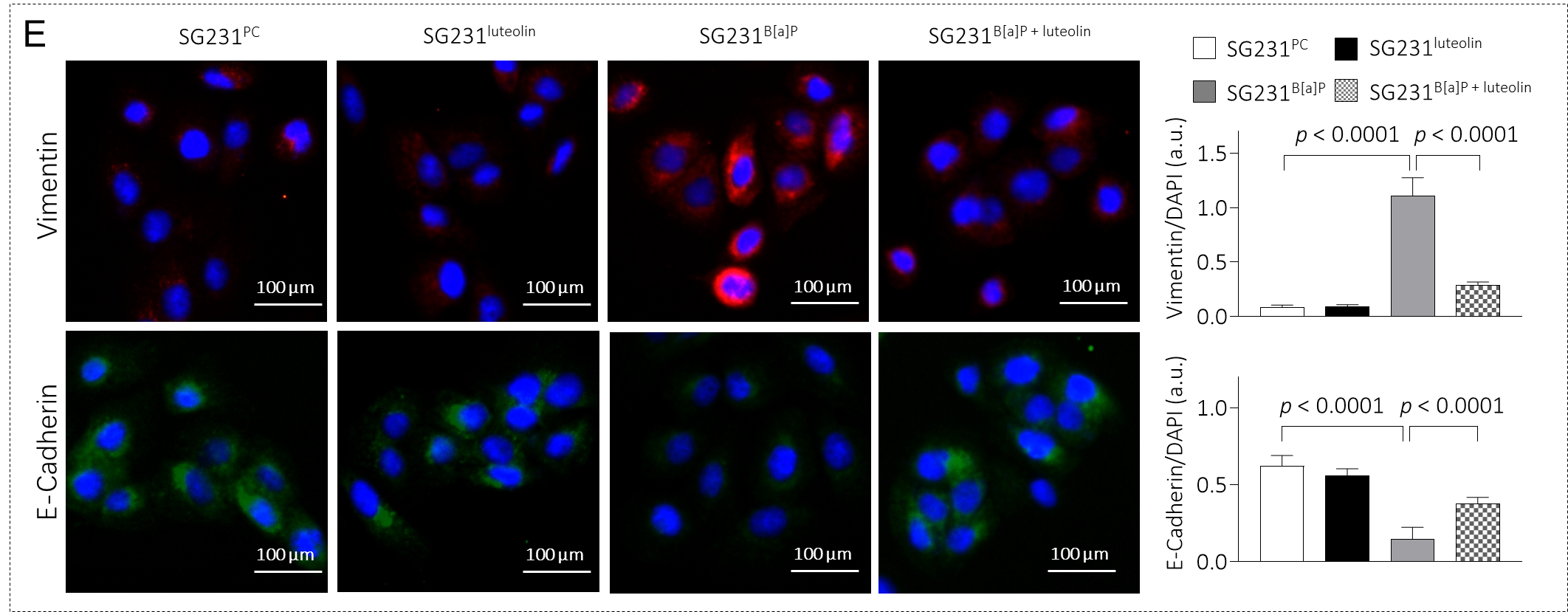


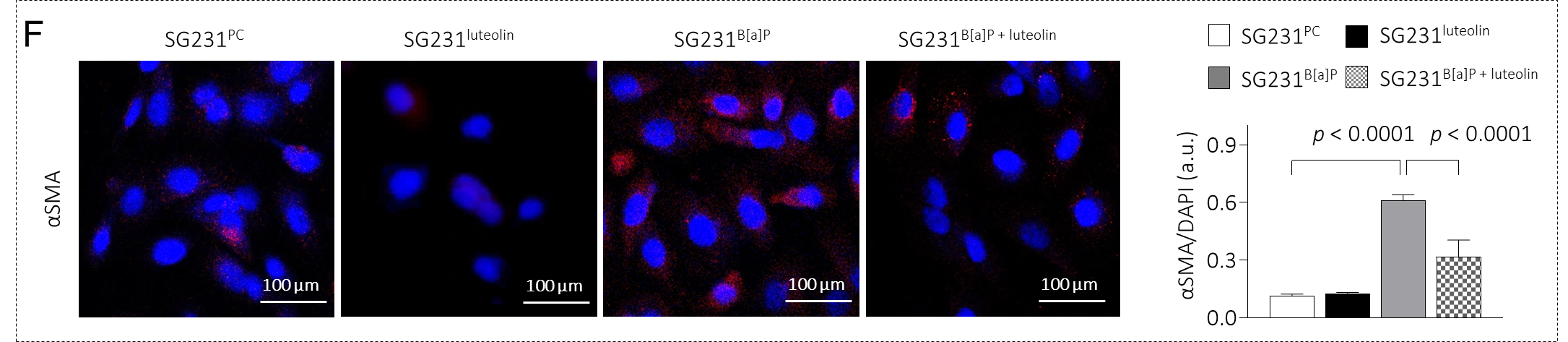


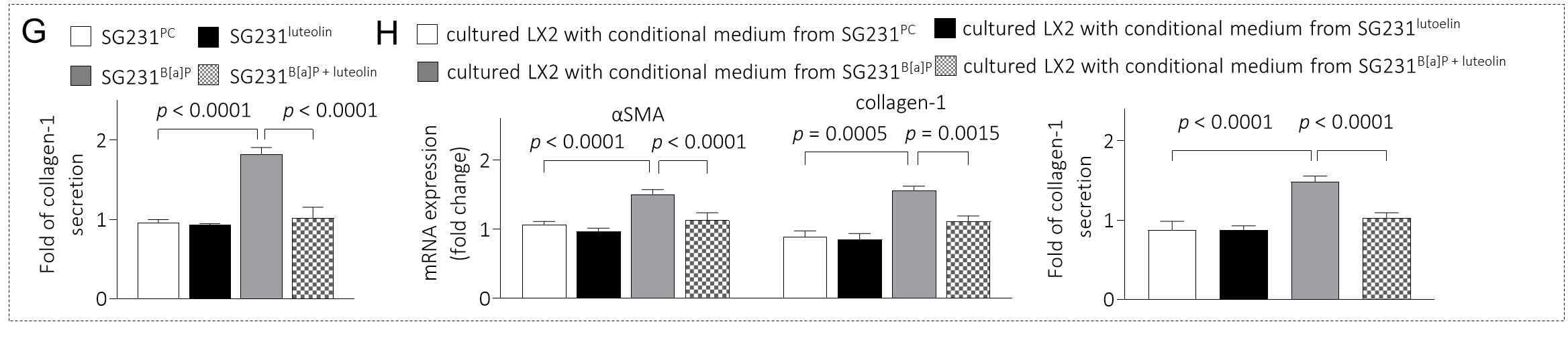


SG231 cells were treated by 0.0 or 100 nM of B[a]P in the presence or absence of 10 μM of luteolin for 6 culture cycles. (A) TEM images. (B) IF staining analysis of mitochondrial and Ca^2+^ co-localisation. (C and D) Intracellular ROS levels. (E and F) IF staining analysis of αSMA, vimentin and E-Cadherin. (G) Triplicate ELISA analysis of collagen-1. (H) After SG231 cells were treated as indicated above, the conditional mediums were collected. LX2 cells were treated by such conditional mediums, triplicate qPCR/ELISA analysis of αSMA and/or collagen-1 were performed. Data was shown as mean ± SD, n =3, an ANOVA followed by Tukey’s t test was used for between-group comparisons.

**Fig. S7. Gender differences in hepatic cirrhosis based on the global burden of disease (GBD) database.**

**
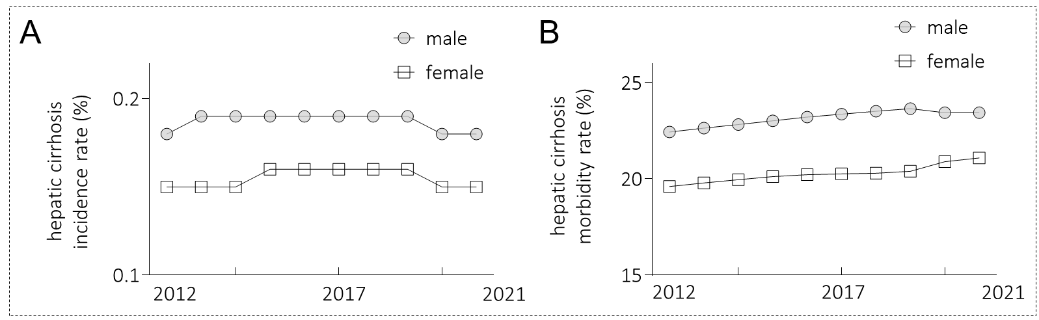
**

Incidence (A) and prevalence (B) of hepatic cirrhosis for men and women based on GBD database from 2012 to 2021. It was found that the prevalence and incidence of hepatic cirrhosis in male population were significantly higher than those in females in the last 10 years. Therefore, we selected 12.5 mg/kg B[a]P for long-term treating the male mice to construct the DR model.

**4. SUPPLEMENTARY REFERENCES**

1. Du X, Jin M, Li R, Zhou F, Sun Y, Mo Q, et al. Mechanisms and targeted reversion/prevention of hepatic fibrosis caused by the non-hereditary toxicity of benzo(a)pyrene. Sci Total Environ 2024, 912: 169496.

2. Yang Y, Jin M, Meng Y, Dai Y, Chen S, Zhou Y, et al. Involvement and targeted intervention of benzo(a)pyrene-regulated apoptosis related proteome modification and muti-drug resistance in hepatocellular carcinoma. Cell Death Dis 2023, 14(4): 265.

3. Jiang F, Li Y, Mu J, Hu C, Zhou M, Wang X, et al. Glabridin inhibits cancer stem cell-like properties of human breast cancer cells: An epigenetic regulation of miR-148a/SMAd2 signaling. Mol Carcinog 2016, 55(5): 929-940.

4. Jin M, Yang Y, Dai Y, Cai R, Wu L, Jiao Y, et al. 27-Hydroxycholesterol is a specific factor in the neoplastic microenvironment of HCC that causes MDR via GRP75 regulation of the redox balance and metabolic reprogramming. Cell Biol Toxicol 2022, 38(2): 311-324.

5. Jin M, Wu L, Chen S, Cai R, Dai Y, Yang H, et al. Arsenic trioxide enhances the chemotherapeutic efficiency of cisplatin in cholangiocarcinoma cells via inhibiting the 14-3-3ε-mediated survival mechanism. Cell Death Discov 2020, 6(1): 92.

6. Shen J, Jiang F, Yang Y, Huang G, Pu F, Liu Q, et al. 14-3-3eta is a novel growth-promoting and angiogenic factor in hepatocellular carcinoma. J Hepatol 2016, 65(5): 953-962.
