# Supplementary figures and images for: Mechanisms and targeted prevention of abnormal ductular reaction caused by a low concentration of Benzo(a)pyrene
Source: Cell Death Dis. 2025 Oct 7;16(1):714. doi: 10.1038/s41419-025-08043-8 (PMC12504665; doi:10.1038/s41419-025-08043-8)

GRP75, 75 kDa

Fig. 2C

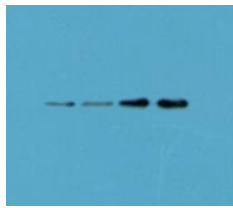

Fig. 4E

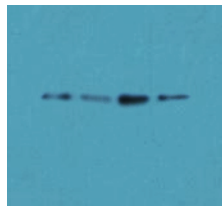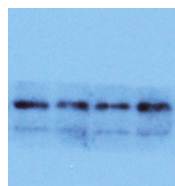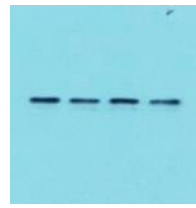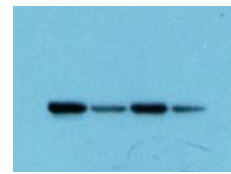

Actin, 45 kDa

Fig. 2C

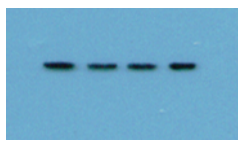

Fig. 4E

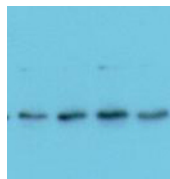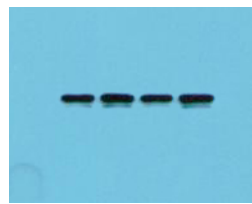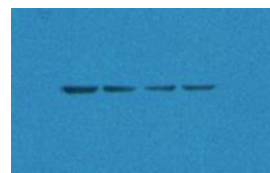

Ub

Fig. 4E

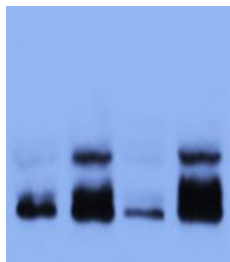

Supplement: Supplementary file 2 — Original western blots [file 41419_2025_8043_MOESM2_ESM.pdf]
